# From optical architectures to actual deployment a review of online process spectroscopy in industrial environments

**DOI:** 10.3389/fchem.2026.1914581

**Published:** 2026-09-04

**Authors:** Huanqing Zhuo, Bo Zeng, Yinghao Ning, Luomeng Zhang, Congcong Tian, Qianyuan Yu, Qian Miao

**Affiliations:** China Tobacco Standardization Research Center, Zhengzhou Tobacco Research Institute of China National Tobacco Corporation, Zhengzhou, China

**Keywords:** acousto-optic tunable filter (AOTF), filter-type structure, fourier transform infrared (FTIR), grating scanning, infrared spectroscopy, online monitoring, optical architectures, process analytical chemistry (PAC)

## Abstract

Real-time quantitative analysis of complex matrices (e.g., moisture and multiple components) is a critical bottleneck in modern Process Analytical Chemistry (PAC). While online near- and mid-infrared spectroscopy are widely deployed, achieving high-fidelity measurements in dynamic industrial environments remains highly challenging. Severe matrix effects coupled with industrial multi-stress interferences, including continuous detector window pollution and light source drive voltage fluctuations, often cause significant baseline drift and non-linear spectral distortion, challenging both physical optical limits and traditional linear calibration models. To address these challenges, this review systematically evaluates the optical architectures and analytical boundaries of four mainstream online infrared spectrometers: filter-based, grating dispersion, Fourier Transform Infrared (FTIR), and Acousto-Optic Tunable Filters (AOTF). Crucially, we comprehensively review the evolution of chemometric compensation strategies designed to overcome these complex spectral interferences. By comparing traditional multivariate linear models, such as Partial Least Squares (PLS), with cutting-edge deep learning frameworks, notably 1D Convolutional Neural Networks (1D-CNN), we discuss the potential of AI-driven soft-calibration for robust feature extraction and precise quantitative prediction under extreme operational stresses. We emphasize that the advantages of such approaches are contingent upon data availability, the degree of spectral nonlinearity, and the specific process environment; they are not a universal replacement for conventional models, which remain effective under stable and linear conditions. Furthermore, the synergistic evolution of miniaturized solid-state hardware is discussed. Ultimately, this review provides a robust theoretical framework for selecting analytical instruments and developing advanced, data-driven chemometric methodologies in dynamic environments, while acknowledging the practical constraints and trade-offs involved in real-world deployment.

## 1 Introduction

In the context of Smart Manufacturing and Industry 4.0, the shift from traditional offline quality control (QC) to online process control and monitoring is imperative. Guided by the Quality by Design (QbD) principle and the Process Analytical Technology (PAT) initiative of the U.S. Food and Drug Administration (FDA), modern industrial production requires standardized data to ensure product stability and reliability (Simon et al., 2015). Online infrared (IR) monitoring has become a key node for information transmission, aiming to ensure product stability and provide standardized data support for quality control. In contrast, traditional offline laboratory analysis relying on manual sampling has significant time lags and cannot meet the strict requirements of real-time feedback and closed-loop control in modern processing (Ismail et al., 2022). Among various PAT tools, Near-Infrared spectroscopy stands out due to its non-destructive nature, non-contact measurement, rapid response, and minimal sample preparation requirements (Chanda et al., 2015; De Beer et al., 2009; Pu et al., 2020). Infrared spectroscopy technology is widely regarded as the preferred sensing technology for online production monitoring, with a wide range of applications including petroleum blending, pharmaceutical fluidized beds, food component analysis, as well as component detection and grading of tobacco and mineral powders (Barla et al., 2014; Berntsson et al., 2002; Bwambok et al., 2020; Chung, 2007; Liu et al., 2024).

The transition from a controlled laboratory environment to a dynamic production line brings numerous challenges, including low resolution under high-speed flow, optical path interference, mechanical vibration, and signal distortion caused by temperature drift (Ferrari et al., 2004). To address these issues, IR instrumentation has evolved through distinct technological routes: from discrete wavelength detection using Filter-type structures, to continuous scanning with Grating spectrometers; followed by FTIR instruments offering high throughput, and finally to AOTF technology designed for harsh environments. These four mainstream architectures differ fundamentally in spectroscopic principles, signal-to-noise ratio (SNR), and response speed, creating specific “ecological niches” suitable for different material types and measurement ranges (Barton and Burdick, 1979; Griffiths et al., 1977; Jacob et al., 1980; Ismail et al., 1997). However, each route faces inherent performance thresholds and bottlenecks regarding resolution, stability, and cost.

Different infrared spectroscopy techniques have their own principles that affect measurement accuracy and are also affected by external factors, resulting in a decline in the stability and reliability of the measurement data. To overcome the aforementioned factors, online IR spectroscopy is undergoing a paradigm shift driven by MEMS, Silicon Photonics, and algorithmic innovations. This shift is transforming spectroscopic instrumentation from bulky mechanical precision structures towards miniaturization and intelligence. Meanwhile, calibration algorithms are evolving from traditional linear regression to deep learning and multi-model transfer learning, partially compensating for hardware-induced spectral distortions (e.g., baseline drift, peak shifts, and scattering effects) through advanced signal processing. However, it should be clarified that these computational methods do not overcome the fundamental physical limits of optical instrumentation, such as diffraction limits, detector sensitivity, or thermal noise floors, but rather serve as an essential complementary layer that maximizes the extraction of chemical information from hardware output (De Graaf et al., 2008; Pasquini, 2018; Wang et al., 2024). It should also be noted that this trajectory does not imply a direct or exclusive transition; a substantial body of intermediate approaches, including adaptive, recursive, incremental, local, hybrid, and other lightweight machine learning methods, has been successfully applied in online industrial spectroscopy, often providing pragmatic solutions under limited data or computational constraints (Ji et al., 2024; Pipanmekaporn et al., 2026; Wu et al., 2022). While these intermediate methods are not the primary focus of this review, their existence provides a more continuous and balanced perspective on the overall methodological evolution.

Therefore, this review provides a systematic overview of the current state of online IR monitoring technologies. We analyze and compare the physical principles, optical architectures, and industrial applications of the four mainstream technologies (Filter, Grating, FTIR, and AOTF). Furthermore, we highlight the emerging advantages of MEMS integration, chip-scale silicon photonics, and deep learning algorithms in achieving dynamic sensing and precision processing, emphasizing that the synergy between miniaturized hardware and intelligent algorithms is the key enabler for next-generation online monitoring systems. Finally, this work offers a theoretical reference for instrumentation selection (decision support) in online process monitoring and discusses future trends towards miniaturization and intelligent manufacturing.

### 1.1 Literature search strategy

To ensure a comprehensive and representative review, relevant publications were collected from major academic databases, including Web of Science Core Collection, Scopus, and Google Scholar. The literature search covered publications from 1977 to 2026 with particular emphasis on recent advances after 2010 in online infrared spectroscopy and process analytical technology.

The search strategy employed combinations of keywords including “online infrared spectroscopy”, “near-infrared spectroscopy”, “mid-infrared spectroscopy”, “process analytical technology”, “chemometric calibration”, “machine learning”, “deep learning”, “adaptive calibration”, and “industrial monitoring”.

Publications were included when they focused on online or in-line infrared spectroscopy for industrial applications, optical instrument architectures, spectral preprocessing, calibration methodologies, or practical deployment issues. Peer-reviewed research articles and representative review papers were considered. Studies limited to laboratory-scale spectroscopy without relevance to process monitoring were excluded.

The selected literature was subsequently organized into three major themes: (i) opti cal architectures of online infrared spectrometers, (ii) chemometric and artificial intelligence-assisted calibration strategies, and (iii) challenges and perspectives associated with industrial deployment.

## 2 Principles and optical architectures of online spectrometers

### 2.1 Filter-based infrared moisture analyzers

**FIGURE 1:**
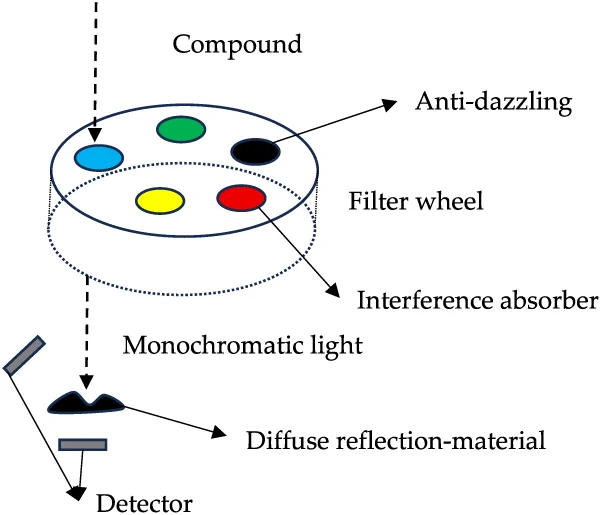
Process diagram of material spectrometry measurement with a filter wheel.

The filter-based infrared moisture analyzer employs a filter wheel equipped with 3–4 narrow-band interference filters acting as splitting elements for specific wavelengths. Driven by a stabilizing motor, the rotation of the filter wheel essentially performs carrier modulation of the optical signal, converting the continuous direct current (DC) light signal into an alternating current (AC) pulse signal, as illustrated in Figure 1. This modulation mechanism allows for the extraction of the effective reflection signal from environmental optical noise using lock-in amplification or high-frequency filtering techniques.

The measurement of moisture content within the infrared spectral region fundamentally relies on the distinct vibrational and rotational energy absorption characteristics of chemical bonds, specifically the H–O bonds in water and C–H bonds in sugars. The core measurement principle is based on the modified Lambert-Beer law (Vera Zambrano et al., 2019). By establishing the absorbance ratio between the measurement wavelength ( $I_{Means}$ ) and the reference wavelength ( $I_{Ref}$ ), common-mode interference is effectively eliminated. As shown in Equation 1, the mathematical model is expressed as follows

$$A=\log\left(\frac{I_{Ref}}{I_{Meas}}\right)\times K$$ (1)

where $I_{Ref}$ and $I_{Meas}$ represent the detector response intensities for the measurement and reference wavelengths, respectively, K is the system constant.

The absorbance value A is calculated from the intensity ratio between the reference and measurement signals. As shown in Equation 2, the moisture content M is calculated using the following equation

**FIGURE 2:**
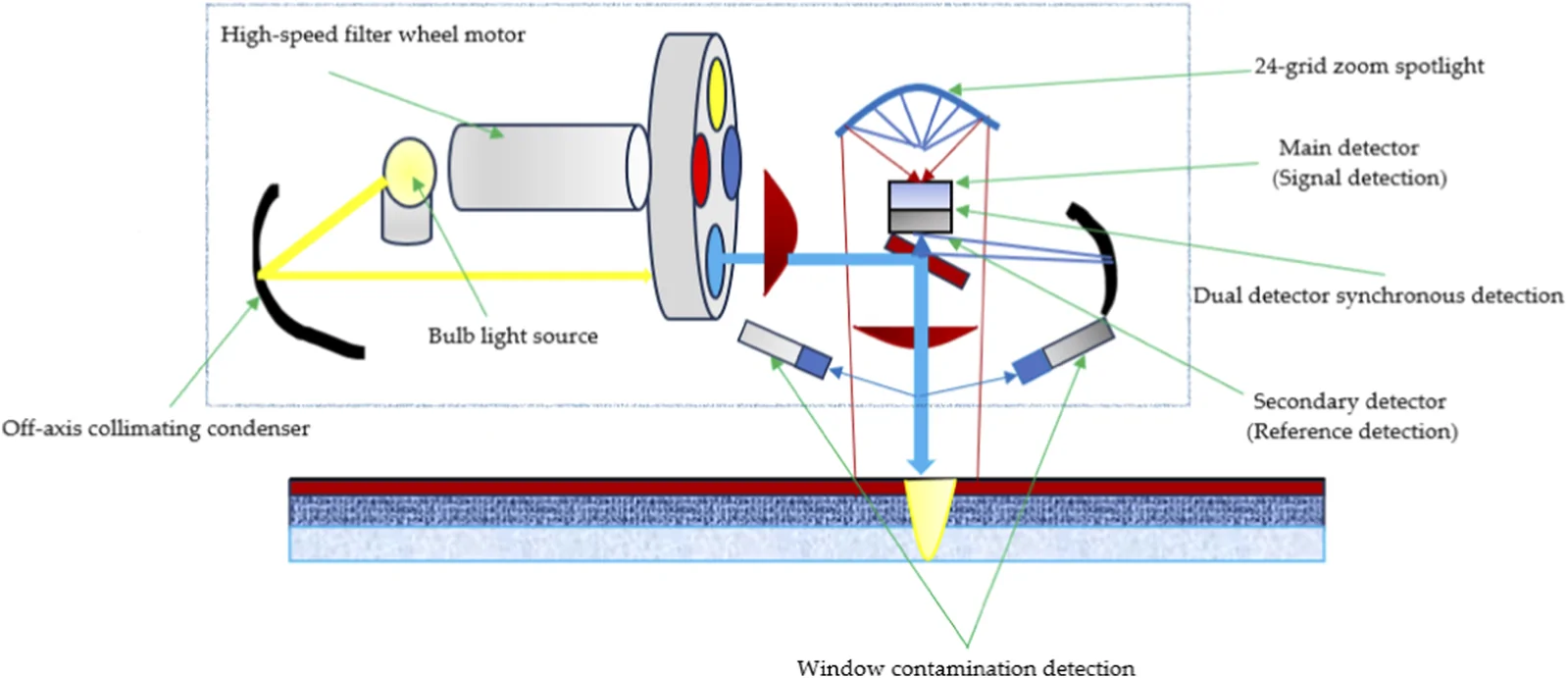
Working principle diagram of a filter-type infrared moisture meter. Reproduced with permission from Ref (Barton and Burdick, 1979).

M=a·A+b (2)

where M represents the predicted moisture content, A represents the absorbance value, a is the calibration slope coefficient, and b is the regression intercept. The workflow of the filter-type infrared moisture analyzer is shown in Figure 2. A tungsten-halogen lamp is typically selected as the stable light source. The light is reflected by an off-axis collimating mirror onto the filters mounted on the rotating wheel, emitting specific wavelengths (e.g., 1.94 μm and 1.45 μm) that are sensitive to H–O bonds. The optical path is designed such that a portion of the light illuminates the measured sample to capture the energy changes before and after reflection, while another portion serves as the reference light. Both signals are absorbed by the detector, and the final moisture value is computed through the algorithm and the light energy absorption model described above (Zhu et al., 2014).

While early filter-based spectrometers primarily relied on a simplified two-wavelength ratiometric model, this architecture has been largely superseded by multispectral designs in modern Process Analytical Technology (PAT). Contemporary industrial filter instruments frequently employ up to six discrete wavelengths to construct robust multivariate calibration models. This evolution is fundamentally driven by the necessity to decouple true chemical absorption from severe physical interferences. By completely eliminating narrow dispersion slits, filter-based architectures achieve maximized optical throughput, consistently delivering an exceptional Signal-to-Noise Ratio (SNR) exceeding 5000:1. Driven by high-speed brushless motors (operating at up to 6000 RPM), these systems accomplish ultra-fast optical modulation. Their response time is mainly determined by detector response, filter switching mechanism, and integration time, achieving transient response times of merely 10–50 ms. In highly heterogeneous streams moving at velocities >2 m/s, these additional wavelengths are strategically utilized to establish dynamic baselines and compensate for complex light scattering effects (Hu, 2021). This exceptional SNR and rapid scanning speed ensure that even exceptionally narrow analytical tolerances are reliably resolved without spectral smearing.

Furthermore, the design paradigm of these discrete-wavelength systems has shifted from hardware-locked configurations to algorithm-driven flexibility. Instead of relying on permanently fixed wavelength sets, modern commercial systems integrate algorithmic selection methods to identify optimal spectral features tailored to specific matrices (Li et al., 2019). Coupled with advanced optical components such as linear variable filters (LVFs), these instruments can be dynamically reconfigured. When quantitatively compared to other spectroscopic principles, filter-based architectures exhibit unparalleled ruggedness: they completely circumvent the mechanical vibration vulnerabilities inherent to Fourier Transform Infrared (FTIR) moving mirrors, and their interference filters maintain a negligible thermal drift coefficient of <0.01 nm/°C, standing in stark contrast to the severe thermal instability (0.15–0.2 nm/°C) characteristic of Acousto-Optic Tunable Filters (AOTF). This software-hardware co-design enables the systems to adaptively mitigate matrix-induced nonlinearities—such as those caused by dynamic window pollution—significantly broadening their applicability and quantitative reliability across diverse and harsh industrial scenarios (Workman and Weyer, 2007).

### 2.2 Grating scanning spectrometers

**FIGURE 3:**
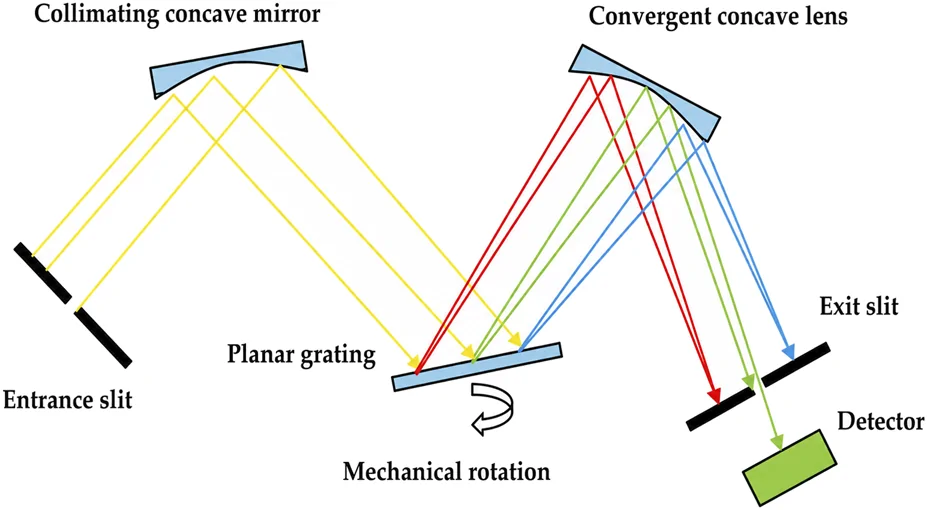
Schematic diagram of the grating mechanical scanning beam splitting principle.

The architectural framework of a grating scanning infrared spectrometer is intrinsically predicated on a precision optomechanical dispersion system. Typically employing a Czerny-Turner or Ebert-Fastie monochromator configuration, the system primarily comprises a broadband high-intensity light source, collimating and focusing optics, a rotatable diffraction grating, and a highly sensitive single-element detector (such as InGaAs or PbS). Functioning as the core high-precision dispersion element, the blazed grating spatially segregates the incident polychromatic beam into its constituent monochromatic wavelengths. By actuating the grating via a precision stepper motor, specific narrow-band wavelengths are sequentially swept across an exit slit to interact with the sample—either in transmission or diffuse reflectance mode—as illustrated in Figure 3. Subsequently, the detector quantifies the optical intensity at each discrete wavelength increment, reconstructing the full spectrum sequentially. As shown in Equation 3, the fundamental principle of this optical dispersion adheres to the grating equation

$$d\left(\sin\theta_{i}+\sin\theta_{d}\right)=m\lambda$$ (3)

where $\theta_{i}$ is the incidence angle, $\theta_{d}$ is the diffraction angle, d is the grating constant (groove spacing), m is the diffraction order, and λ is the wavelength. This equation establishes a strict mapping relationship between the spatial angle and the wavelength, allowing the instrument to scan across the spectrum by precisely controlling the rotation angle of the grating. To further characterize the theoretical limit of the instrument’s performance. As shown in Equations 4–5, the angular dispersion ( D ) and the theoretical resolving power ( R ) are introduced

$$D=\frac{d\theta_{d}}{d\lambda }=\frac{m}{dcos\theta_{d}}$$ (4)

$$R=\frac{\lambda }{\Delta \lambda }=mN$$ (5)

where N is the total number of illuminated grooves. These theoretical derivations indi cate that the spectral resolution of the system is jointly determined by the physical dimensions of the grating, the groove density, and the mechanically defined physical width of the incident and exit slits.

**FIGURE 4:**
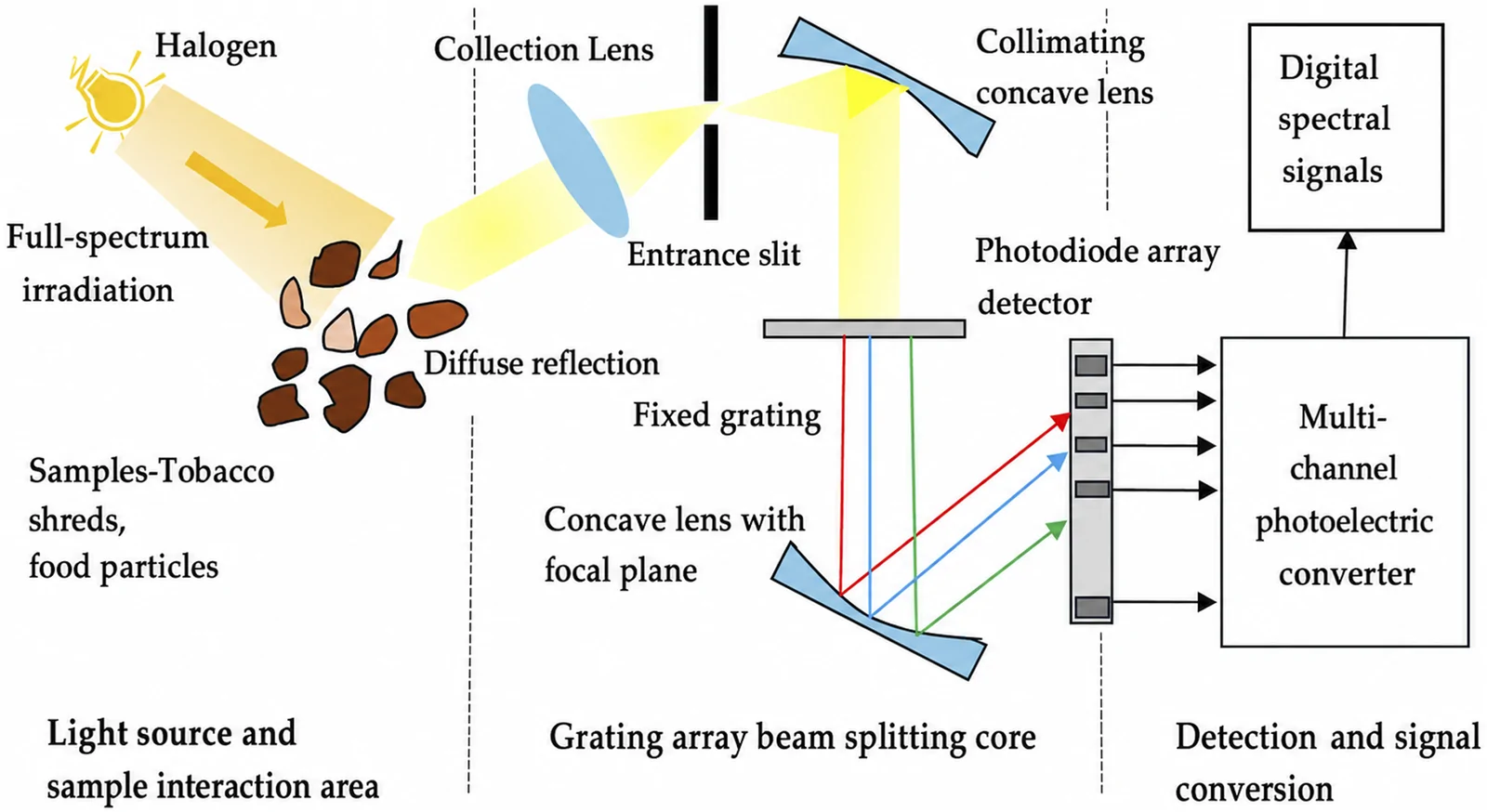
Array spectrometer component measurement process diagram.

From an analytical perspective, high spectral resolution is a primary advantage of grating scanning spectrometry. In contrast to the discrete wavelength measurements characteristic of filter-based architectures, grating dispersion units generate continuous spectra that span the entire near-infrared region, typically ranging from 800 to 2500 nm, as shown in Figure 4. This full-wavelength-coverage spectrum can better distinguish overlapping absorption peaks, thus enabling the measurement of complex multi-component matrices, including water, sugars, nicotine, and amino acids (Bagusat et al., 2019). Grating scanning infrared spectrometer acquires spectra through sequential wavelength scanning. Their acquisition speed depends on the scanning range, mechanical scanning speed, spectral resolution, and required signal-to-noise ratio. For industrial monitoring, full-spectrum acquisition generally requires seconds, although optimized systems can achieve faster measurements with reduced spectral resolution. While the grating equation and resolving power formulas define the theoretical optical limits of dispersive instruments, their practical deployment in online monitoring is fundamentally constrained by an unavoidable “resolution-throughput-speed” trade-off. For instance, configuring a near-infrared grating spectrometer for high spectral resolution (e.g., 2 nm) necessitates extremely narrow entrance and exit slits (typically 10 to 25 um ). However, this drastic reduction in optical throughput (etendue) severely limits photon collection. To maintain a baseline Signal-to-Noise Ratio (SNR) of 5000:1 under such low-light conditions, the detector requires significantly longer integration times, often exceeding 500 milliseconds.

This physical limitation makes high-resolution dispersive systems fundamentally income patible with the rapid online analysis of high-speed, heterogeneous moving materials, such as tobacco shreds or mineral powders traveling on a conveyor belt at >2 m/s. At such velocities, a 500 ms integration time would result in severe spatiotemporal misalignment and spectral smearing. Consequently, in Process Analytical Technology (PAT), grating instruments are typically optimized by physically widening the slits to 100–200 um This hardware modification maximizes optical throughput and compresses the required integration time to merely 5–10 ms, enabling ultra-fast scan rates of up to 100 Hz. Although this strategy deliberately sacrifices spectral resolution (degrading to 10–15 nm), it ensures a robust SNR (>2000:1) and the real-time, blur-free capture of dynamic matrix variations (Bower, 1992; Workman and Weyer, 2007).

### 2.3 Fourier transform infrared (FTIR) spectrometers

**FIGURE 5:**
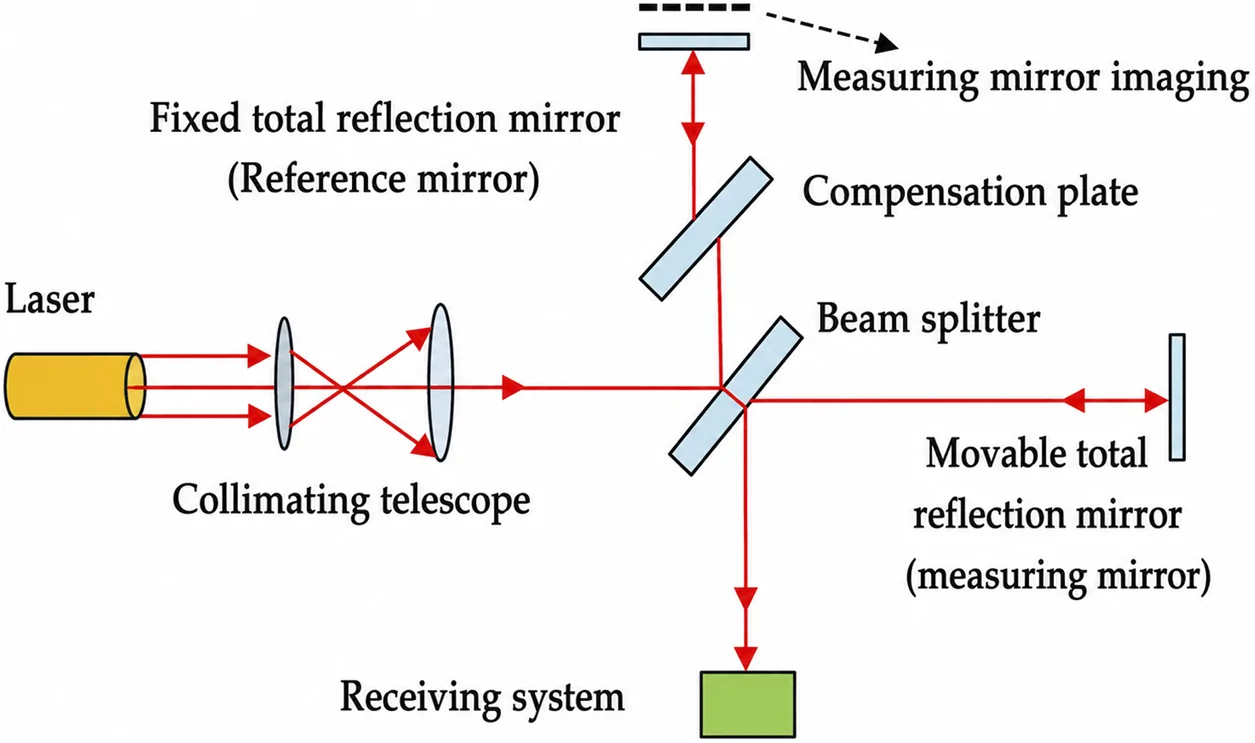
Michelson interferometer optical path. Adapted with permission from Ref (Linares-Herrero et al., 2012; Yuan et al., 2023).

Diverging from filter-type and grating scanning architectures that are predicated on dispersive elements, FTIR spectrometers employ a Michelson interferometer as the central optical component, thereby acquiring spectral information through the precise modulation of the Optical Path Difference (OPD). As illustrated in Figure 5, the fundamental workflow involves a broadband infrared light source emitting radiation that passes through a collimating lens into a beam splitter. The beam splitter divides the optical path into two beams: one is reflected towards a fixed mirror, while the other is transmitted towards a moving mirror. The moving mirror translates back and forth at a constant velocity to generate a varying OPD (Watari, 2010).

Data acquisition primarily involves the detector recording an interferogram, which represents the light intensity curve as a function of the moving mirror’s position, rather than direct spectral information. As shown in Equation 6, for monochromatic light with a wavelength λ (cm), the corresponding wavenumber v (cm^-1^) is defined as

$$v=\frac{1}{\lambda }$$ (6)

As shown in Equation 7, the phase difference δ, between the two coherent beams is determined by the OPD x

δ=2πvx (7)

As shown in Equation 8, for the interference formed by ideally collimated polychromatic light, the intensity formula is

$$I\left(x,v\right)=\int_{-\infty }^{+\infty }S\left(v\right)\cos\left(2\pi vx\right)dv$$ (8)

where I is the interference intensity and $S\left(v\right)$ is the spectral intensity distribution of the incident light. To convert the time-domain interferogram into a frequency-domain spectrum, As shown in Equation 9, an Inverse Fourier Transform is applied

$$S\left(v\right)=\int_{-\infty }^{+\infty }I\left(x\right)e^{-i2\pi vx}dx$$ (9)

Through the mathematical relationship between spectral intensity $S\left(v\right)$ , interference intensity $I\left(x,v\right)$ , and OPD x , the curve correlating absorbance with wavenumber is obtained. Since the interferogram signal is analog, it must undergo discrete sampling. To prevent spectral aliasing and data loss. As shown in Equation 10, the sampling interval $\Delta_{x}$ and the wavenumber range $v_{\min}∼v_{\max}$ must satisfy the Nyquist sampling theorem

$$\Delta_{x}\leq \frac{1}{2\left(v_{\max}-v_{\min}\right)}$$ (10)

**FIGURE 6:**
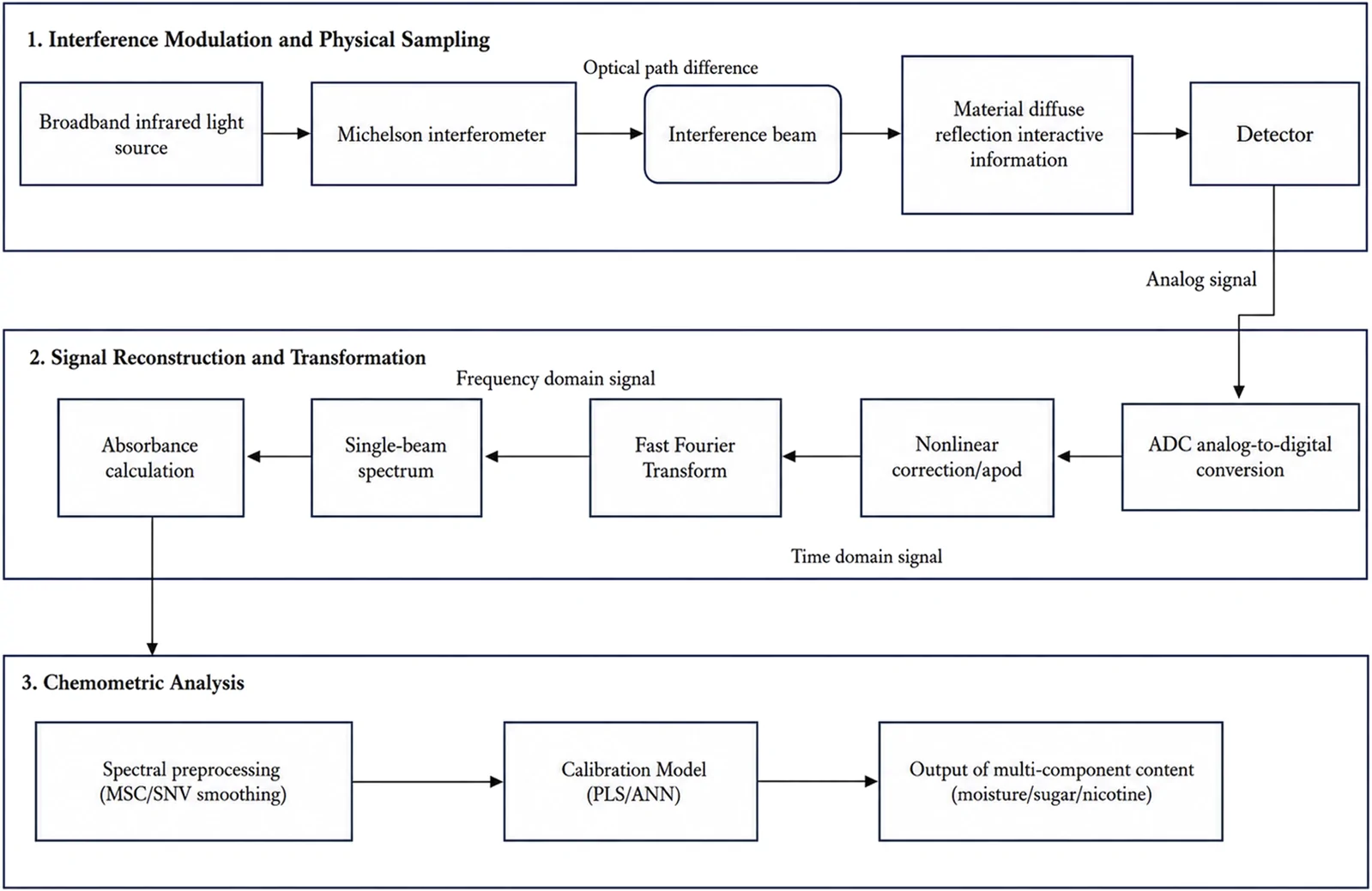
Fourier transform infrared spectrometer workflow.

Subsequently, the absorbance spectrum is calculated by comparing the single-beam spectrum of the sample against the background spectrum. The computed spectrum undergoes preprocessing to eliminate baseline drift and particle scattering, followed by the application of Chemometrics or machine learning models, which are employed to quantify moisture and sugar content by analyzing spectral intensity variations (Yuan et al., 2023). The workflow of the FTIR spectrometer is shown in Figure 6.

From an instrumental performance perspective, FTIR serves as the standard paradigm for high-precision and multi-component measurement. Compared to dispersive instruments, it possesses three distinct advantages. First, FTIR spectrometers do not require entrance or exit slits, their optical throughput (etendue) is typically 100 to 200 times higher than that of a dispersive grating spectrometer operating at an equivalent spectral resolution. Secondly, the multiple channels of an FTIR detector can be reused repeatedly with different characteristic light waves. The detector can simultaneously receive signals of all wavelengths, making FTIR measurements 10 to 100 times faster than grating scanning. Additionally, the use of a He-Ne laser for internal calibration ensures high wavenumber precision (up to 0.01 cm^-1^), improving the reliability and accuracy of the calibration model and establishing a baseline for accuracy calibration between different spectrometers.

While FTIR mathematically satisfies the Nyquist criterion for high-precision interferogram sampling, the practical acquisition time is influenced by spectral resolution, optical path difference scanning speed, detector performance, and the number of averaged scans. Typical industrial measurements may range from sub-second acquisition to several seconds depending on analytical requirements. Its deployment in online process monitoring is fundamentally constrained by the high sensitivity of moving mirror interferometers to mechanical vibration and thermal drift. In a standard Michelson interferometer, the moving mirror must maintain strict orthogonality during translation. Vibrational perturbations causing angular tilts as small as a few microradians (typically>1 × 10^−5^ rad) can severely degrade the modulation efficiency and fringe contrast, rendering the resulting spectral data completely unusable for quantitative analysis.

To mitigate these pervasive industrial interferences, modern online FTIR systems employ sophisticated optical engineering strategies. One primary approach is dynamic alignment, which utilizes a reference HeNe laser to continuously monitor the optical path error and drives piezoelectric actuators at kilohertz frequencies to correct the orientation of the fixed mirror in real time. Alternatively, many robust industrial systems replace flat mirrors with corner-cube retroreflectors. Because corner cubes inherently reflect light perfectly parallel to the incident beam regardless of their exact orientation, they provide intrinsic optical immunity to mechanical tilt (pitch and yaw) induced by plant floor vibrations (Linares-Herrero et al., 2012). Through these engineering interventions, FTIR can be successfully deployed in continuous manufacturing environments, though it still fundamentally prefers environments with controlled mechanical stress compared to solid-state sensors (Smith, 2011).

### 2.4 Acousto-optic tunable filter (AOTF) spectrometers

The AOTF spectrometer represents a paradigm shift in process analysis technology from mechanical scanning to solid-state instruments. In contrast to conventional grating or filter-based architectures that depend on mechanical rotation, AOTF technology operates as a solid-state system devoid of moving parts. This intrinsic structural stability eliminates measurement aberrations induced by vibration and mechanical wear, thereby establishing it as a highly reliable solution for complex operational environments. Its fundamental capability lies in high-speed electronic tuning and random wavelength access, which facilitates the discrete scanning of arbitrary wavelengths.

**FIGURE 7:**
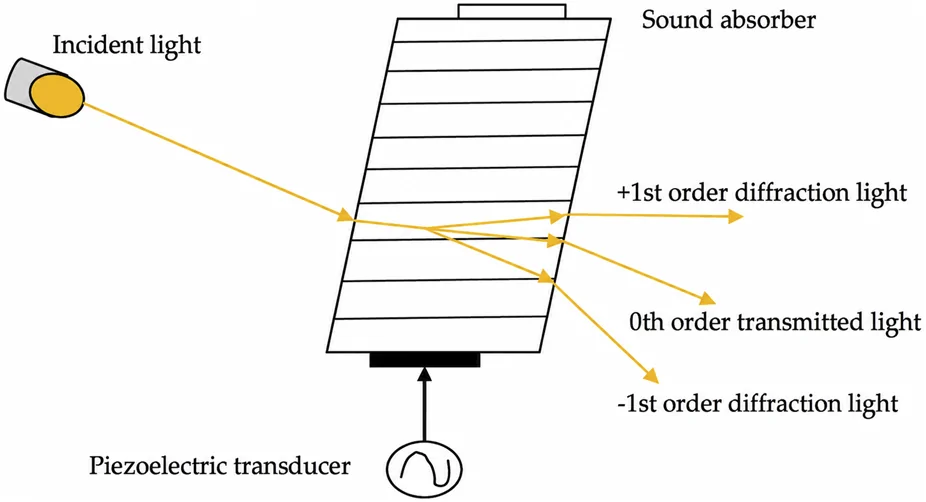
AOTF structural diagram. Adapted with permission from Ref (Bei, 2004; Coffey et al., 1998; Gupta, 2002; Mi et al., 2024).

Functionally, the AOTF constitutes a solid-state dispersive element based on the acousto-optic diffraction effect. The core components of an AOTF assembly typically comprise a birefringent Tellurium Dioxide (TeO_2_) crystal bonded to a piezoelectric transducer and a Radio Frequency (RF) driver. The RF driver generates a high-frequency electrical signal (ranging from 30 to 200 MHz), which is applied to the transducer. The transducer converts this electrical energy into an ultrasonic wave propagating within the crystal. This ultrasonic field induces periodic elastic deformation inside the crystal lattice, causing a periodic variation in the refractive index. This variation establishes an equivalent moving phase grating optically. When broadband infrared light is incident upon the crystal, specific wavelengths that satisfy the momentum matching condition are selectively diffracted and separated from the polychromatic beam (Bei, 2004). The structural schematic is shown in Figure 7.

As shown in Equation 11, the tuning relationship satisfying the momentum matching condition is expressed as

$$\lambda =\Delta n\cdot \alpha \cdot \frac{v_{a}}{f_{a}}$$ (11)

where λ is the diffracted central wavelength, Δn is the birefringence difference of the crystal ( $n_{e}-n_{o}$ ), $v_{a}$ is the acoustic velocity within the crystal, and $f_{a}$ is the applied RF frequency. The wavelength λ is inversely proportional to the RF frequency $f_{a}$ . By altering the frequency of the electrical signal, the filtered optical wavelength can be switched within microseconds, a speed determined solely by the acoustic transit time across the optical aperture.

**FIGURE 8:**
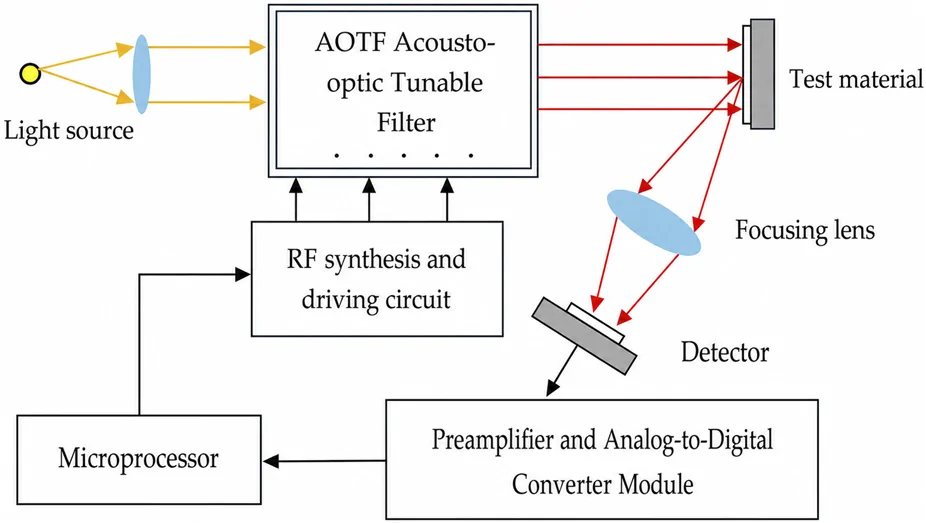
Closed-loop operation of an acousto-optic tunable filter for measuring material composition.

AOTF technology preserves measurement accuracy and stability by integrating a dual-beam reference optical path and implementing closed-loop feedback to regulate the RF signal amplitude. The diffracted beam serves as the measurement beam, while the zero-order undiffracted light (or a beam-split portion) serves as the reference to monitor source fluctuations in real-time. Furthermore, the RF signal undergoes amplitude modulation, causing the detector to output an Alternating Current (AC) signal. This allows the circuit to employ synchronous demodulation and lock-in amplification techniques to effectively isolate the target signal from environmental background noise (Gupta, 2002). The closed-loop control process of the AOTF is illustrated in Figure 8.

While the all-solid-state design and random wavelength access of Acousto-Optic Tunable Filters (AOTF) provide exceptional scan speeds, these advantages entail significant analytical trade-offs. First, at a 50 Hz scanning rate, the optical integration time for each individual wavelength channel can be reduced to below 20 ms. However, the overall spectral acquisition time depends on the number of selected wavelengths, spectral range, spectral resolution, and signal averaging requirements. According to the square-root law of signal averaging, this rapid operational speed inherently results in a Signal-to-Noise Ratio (SNR) degradation of approximately 60%–80% compared to standard static scanning modes (e.g., 1 s integration).

Secondly, their wavelength tuning stability is inherently compromised by the thermal sensitivity of the TeO_2_ crystal. In the near-infrared region, the thermal drift coefficient of acoustic birefringence in AOTFs is typically around 0.15–0.2 nm/ °C, which is in stark contrast to traditional interference filters that exhibit exceptional thermal stability (typically <0.01 nm/ °C). Without precision Thermoelectric Cooling (TEC), a mere 5 °C ambient fluctuation on the factory floor can induce a 1 nm peak shift. Consequently, in real-world process analytical applications—such as the continuous in-line monitoring of moisture dynamics in agricultural processing or the tracking of active pharmaceutical ingredients within fluid bed dryers (Coffey et al., 1998), where ambient and process-induced temperatures routinely fluctuate by 5–10 °C—the filtered wavelength can shift by 1–2 nm. As demonstrated in recent literature (Mi et al., 2024; Brimrose Corporation, 2021), this magnitude of drift is catastrophic when targeting narrow absorption features or applying derivative preprocessing to sharp moisture overtones (e.g., the 1,450 nm and 1940 nm bands). Ultimately, these combined penalties of severe SNR degradation and thermal instability explain why advanced chemometric compensation is strictly required for robust online AOTF deployments.

### 2.5 Comparative analysis and selection guide

Driven by the dual demands for detection precision and real-time responsiveness, infrared monitoring technology has evolved from early single-parameter moisture analyzers to multi-component online analysis systems covering complex matrices such as sugar and nicotine (Xin et al., 2014; Zhu et al., 2022). The optical structure and characteristic wavelength extraction of near-infrared spectrometers have historically limited measurement accuracy and real-time feedback. Traditional mechanical spectrometers are often constrained by a trade-off between high sensitivity at specific wavelengths and diminished resolution elsewhere, a limitation that restricts instrument versatility and impedes multi-component quantification. Conversely, solid-state electronic architectures, such as acousto-optic modulators and MEMS, surmount the inherent deficits of discrete-wavelength instrumentation. By exploiting multi-channel architectures and random access capabilities, these technologies facilitate comprehensive full-spectrum and multi-wavelength analysis. Furthermore, they accelerate the system’s transient response to the microsecond regime, thereby substantially broadening the applicability of process analytical techniques.

The optimal selection of an infrared spectrometer necessitates a holistic evaluation that extends beyond optical architectures and fundamental operating mechanisms to encompass strategic engineering trade-offs regarding resolution, temporal response, measurement fidelity, and economic constraints. Traditional near-infrared spectrometers—including filter-type, grating-based, FTIR, and AOTF systems—predominantly rely on mechanical-optical architectures. These configurations are inherently susceptible to vibration and noise, which can compromise resolution and the extraction of characteristic wavelength information. Conversely, contemporary advancements are pivoting towards static, multispectral analysis capable of resolving complex data. As optical structures become increasingly miniaturized and integrated with silicon photonics, modern systems demonstrate the spectral resolution necessary to discern minute differences in absorption peaks. Crucially, this selection process must be aligned with the specific exigencies of the *in situ* operational environment and the requisite analytical precision for the target composition. Synthesizing the operational principles and performance metrics detailed in the preceding analysis, Table 1 provides a comprehensive comparative summary.

1. For single-component monitoring applications, filter-type architectures constitute the optimal solution. Their operational mechanism facilitates the extraction of high-fidelity data by quantifying differential energy absorption at discrete wavelengths. Characterized by a streamlined optical configuration and efficient signal processing, these spectrometers exhibit superior thermal dissipation and stability. Consequently, they have become the predominant choice for moisture monitoring in mineral powder and cigarette manufacturing, particularly in scenarios defined by distinct target absorption peaks and minimal spectral interference (Wang, 2024; Yu and Zheng, 2019).
2. Grating scanning spectrometers are extensively utilized in dynamic processes necessitating multi-component quantification, owing to their capacity for full-spectrum acquisition and complex data processing. Characterized by balanced resolution and response speeds, their mechanical scanning mechanism facilitates the extraction of spectral features rich in compositional information. Thus, they are particularly well-suited for sectors such as agriculture and food processing, where comprehensive spectral datasets are a prerequisite for robust chemometric modeling (Wilkes et al., 2024).
3. For comprehensive multi-component analysis in controlled offline environments, Fourier Transform Infrared (FTIR) spectroscopy constitutes the preeminent analytical methodology. Predicated on interferometric modulation, it leverages the multiplex (Fellgett) and high throughput (Jacquinot) advantages to establish a rigorous measurement benchmark. The technology affords superior spectral resolution and precision—capable of quantifying moisture content to within ±0.05%—rendering it ideal for validation and pilot testing in the petrochemical and pharmaceutical sectors. However, its inherent sensitivity to mechanical perturbations necessitates stringent vibration isolation, thereby constraining its direct utility in high-noise, high-vibration industrial settings (Hellwig et al., 2025; Čolnik et al., 2021).
4. The high-speed closed-loop control mechanism inherent to AOTF systems ensures rapid response and random spectral access, even under adverse operating conditions. By precisely modulating the frequency of the electronic drive signal, the system selectively tunes specific optical wavelengths for targeted data acquisition. Furthermore, its solid-state topology and microsecond-scale temporal resolution (≤10 ms) facilitate the real-time monitoring of rapid material flux within pipelines or confined geometries without mechanical degradation. While this architecture guarantees superior operational stability and efficiency, it entails a correspondingly higher capital investment, such as narrow pharmaceutical V-blenders and micro-tubular continuous-flow reactors (Yu et al., 2023; Yue et al., 2012).

**TABLE 1:** Comparison of spectral modes and performance parameters of four infrared spectrometers.

Architecture	Spectral mode	Resolution/Accuracy	Intrinsic Response time	Full-spectrum acquisition time	Main factors affecting speed	Application
Filter-based	Discrete (Fixed Points)	Moisture: ±0.1%–0.3%; Organics: N/A	10–50 ms (selected wavelength measurement)	N/A	Detector response, filter switching mechanism, integration time, number of selected wavelengths	Tobacco shreds (Wang, 2024; Yu and Zheng, 2019)
Grating Scanning	Continuous (Full Spectrum)	Moisture: ±0.1% Organics: ±0.2%–0.5%	ms-level detector response	1–10 s	Scanning range, grating movement speed, spectral resolution, signal averaging	Food pastes or polymer melts (Wilkes et al., 2024)
FTIR	Continuous (Full Spectrum)	Moisture: ≤0.05% Organics: ±0.1%	ms-level detector response	1–10 s	Optical path difference, mirror velocity, spectral resolution, signal averaging requirements	pharmaceutical syntheses (Hellwig et al., 2025; Čolnik et al., 2021)
AOTF	Discrete/Random Access	Moisture: ±0.1% Organics: ±0.2%–0.4%	us-level wavelength switching capability	0.01– several seconds	Number of selected wavelengths, spectral range, resolution, detector integration time, averaging requirements	micro-tubular continuous-flow reactors (Yu et al., 2023; Yue et al., 2012)

## 3 Signal processing and chemometric strategies in complex matrices

**FIGURE 9:**
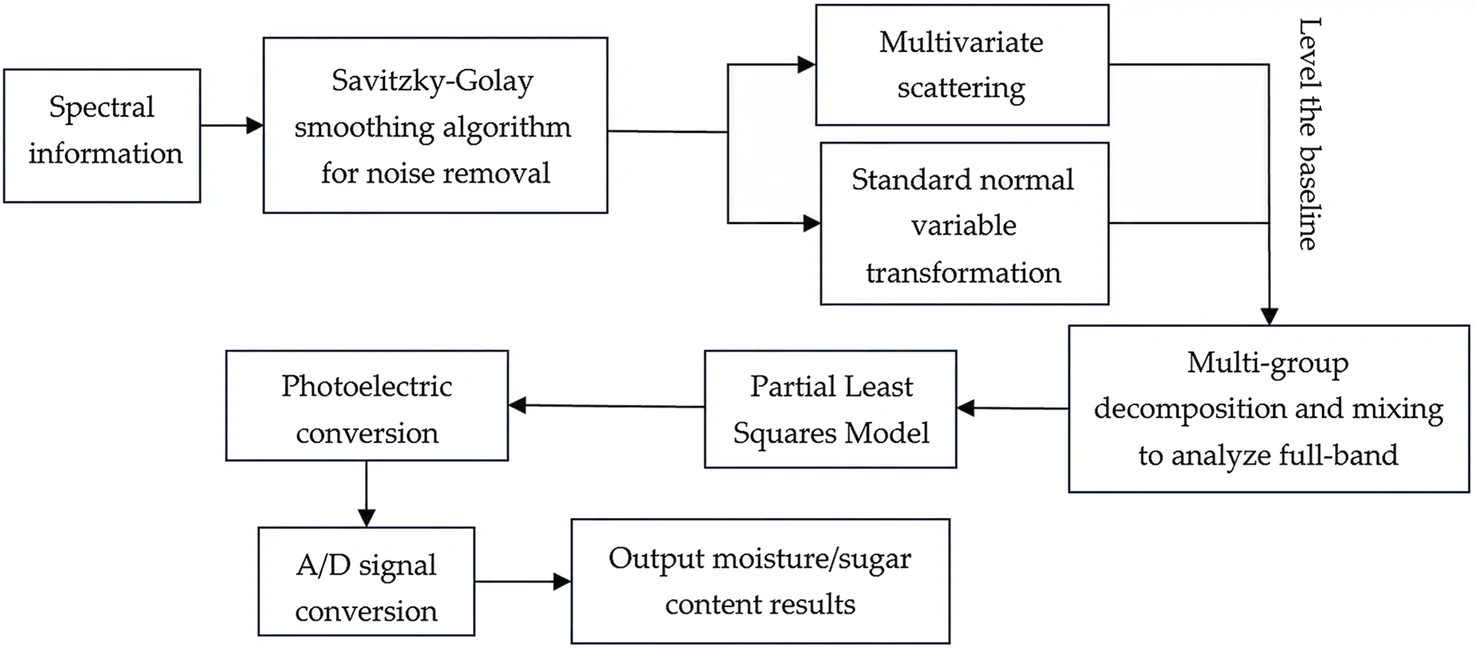
The process of chemometric analysis of material composition and signal conversion.

The acquisition and analysis of spectral data regarding infrared energy variations constitute a fundamental prerequisite for online process control; the development of the associated algorithmic models encapsulates the analytical characterization of these specific fluctuations in spectral energy. In industrial production environments, raw spectral information is inevitably influenced by ambient temperature and humidity, as well as the intrinsic color and morphology of the material (Chu et al., 2022). These factors induce light scattering and spectral baseline drift, subsequently causing deviations in the measurement data. While linear and regression models can theoretically correct for light scattering and baseline offsets, industrial processes are subject to unquantifiable factors such as mechanical vibrations, dust accumulation, and variability in manual operations, all of which compromise the accuracy of the final data. Furthermore, given that measurement materials typically contain diverse chemical components, single-parameter monitoring is evidently insufficient to meet modern production standards. There is a critical need for multi-parameter measurement within complex matrices, a task for which single-algorithm models are often inadequate. Consequently, optimizing chemometric strategies and evaluating anti-interference capabilities through the localized deployment of algorithms is a practically feasible approach. Figure 9 illustrates the workflow for the chemometric resolution of material composition and signal conversion. Given the susceptibility of traditional linear models to spectral misalignment, external perturbations, and the inherent complexity of material matrices, it is imperative to transition the algorithmic framework from conventional linear regression toward advanced machine learning and deep learning architectures.

### 3.1 Spectral preprocessing process and algorithm

Spectral measurements are inherently susceptible to significant background noise and baseline drift, necessitating the employment of algorithmic models for effective correction and denoising. The acquired spectral information often contains substantial background noise due to multifactorial influences such as environmental temperature and humidity, mechanical vibration, and the internal structure of the instrument. These factors inevitably lead to baseline drift and light scattering, necessitating the application of algorithmic models to denoise the spectral background and correct for baseline and scattering effects (Jiao et al., 2020; Zhang et al., 2022; Zhu et al., 2024).

In the preprocessing stage, the Savitzky-Golay (SG) smoothing method is universally employed to remove random electronic noise and enhance the SNR while preserving the original spectral waveform. This method is particularly applicable in environments characterized by weak signals and high detector noise. As shown in Equation 12, its mathematical expression is

$$x_{i}^{*}=\frac{\sum_{j=-m}^{m}c_{j}x_{i+j}}{N}$$ (12)

where $x_{i}^{*}$ is the smoothed value, 2m+1 is the window size, $c_{j}$ represents the polynomial convolution coefficients, and N is the normalization factor.

Following noise reduction, derivative techniques are adopted to correct baseline drift caused by temperature variations and light source aging. The first derivative effectively eliminates constant baseline offsets, whereas the second derivative separates overlapping absorption peaks after removing linear baseline tilts. This capability is of significant importance for the accurate component measurement of complex mixed materials.

To accommodate diverse operational conditions and inherent variations in material composition, sole reliance on derivative-based correction and noise reduction proves inadequate for complex *in situ* environments. Consequently, the implementation of advanced preprocessing algorithms, notably Standard Normal Variate (SNV) and Multiplicative Scatter Correction (MSC), is requisite for robust data processing in industrial settings. SNV standardizes each spectrum individually without requiring a reference spectrum, making the SNV algorithm highly suitable for measurement during industrial production processes. Conversely, MSC requires a reference spectrum for regression correction, resulting in lower adaptability for online material component measurement.

As shown in Equation 13, the mathematical expression for Standard Normal Variate (SNV) is

$$x_{SNV}=\frac{x_{i}-\bar{x}}{\sigma }$$ (13)

where $\bar{x}$ is the mean and σ is the standard deviation.

As shown in Equations 14–15, Multiplicative Scatter Correction (MSC) corrects scattering by performing a regression between the measured spectrum $\left(x_{i}\right)$ and an ideal reference spectrum ( ${\bar{x}}_{ref}$ ), mathematically expressed as

$$x=a+b\cdot {\bar{x}}_{ref}+e$$ (14)

$$x_{MSC}=\frac{x_{i}-a}{b}$$ (15)

where a is the intercept and b is the slope (scattering coefficient).

While SNV and MSC are ubiquitous for baseline correction, treating them as universally applicable solutions for light scattering represents a significant oversimplification in Process Analytical Technology (PAT). Both algorithms implicitly assume that physical scattering manifests purely as independent additive and multiplicative effects. However, in highly absorbing or strongly scattering industrial matrices (such as high-moisture agricultural streams or dense pharmaceutical powders), this assumption frequently breaks down. Critically, when physical scattering phenomena (e.g., particle size aggregation) are highly correlated with the target analyte’s concentration (e.g., moisture content), indiscriminately applying SNV or MSC can inadvertently strip away chemically relevant variance, thereby degrading rather than improving the predictive model (Mallet et al., 2021).

To address these fundamental limitations, modern chemometric workflows increasingly rely on more sophisticated signal processing alternatives. Extended Multiplicative Signal Correction (EMSC) advances beyond simple MSC by incorporating polynomial baselines and known physical interference spectra directly into the correction model, allowing for the effective decoupling of chemical absorption from severe physical scattering (Tafintseva et al., 2021). Alternatively, External Parameter Orthogonalization (EPO) offers a robust mathematical framework that identifies the subspace of parasitic variations (such as uninformative scattering, uncalibrated sensor drift, or thermal fluctuations) and projects the spectral data orthogonally to this subspace. By isolating and removing only the disruptive physical variance, EPO perfectly preserves the pure chemical signal required for robust online monitoring (Gamagedara et al., 2025).

Preprocessing yields reliable data for modeling and deployment. SG smoothing reduces noise but may weaken weak signals; derivative sharpens resolution but amplifies noise. SNV and MSC efficiently correct scattering, yet their additive/multiplicative assumptions often fail for complex samples. Advanced methods like EMSC and EPO are more robust by accounting for physical interferences, but they require more prior knowledge and tuning. Thus, preprocessing should be tailored to spectral characteristics and interference sources, not treated as universal, to improve robustness and practical applicability.

### 3.2 Evolution of online infrared monitoring calibration mode

Calibration methods for online infrared spectroscopy should not be interpreted as a simple progression from linear chemometrics to increasingly complex artificial intelligence models. Their suitability depends on the relationship between spectral responses and target properties, the magnitude and type of process disturbances, the size and representativeness of the operational requirements of the intended deployment environment. Linear chemometric, nonlinear machine-learning, deep-learning, and adaptive calibration approaches therefore represent complementary rather than strictly successive modeling paradigms.

When spectral variation is approximately linear and the calibration domain is adequately represented, Partial Least Squares regression remains an effective choice because of its computational efficiency, interpretability, and robustness under limited-data conditions. In contrast, nonlinear machine-learning models may be advantageous when moderate nonlinear relationships remain after preprocessing. Deep-learning models can further learn hierarchical spectral features when sufficiently large and representative datasets are available, but their higher model complexity also increases the risks of overfitting, data leakage, and poor transferability. For long-term industrial deployment, adaptive updating and calibration-transfer strategies may be more important than increasing model complexity alone.

As the standard paradigm in chemometrics, PLS addresses the multicollinearity problem inherent in high-dimensional spectral data by projecting both predictor and response variables into a low-dimensional latent space. Distinct from standard regression paradigms, PLS synthesizes spectral predictors and chemical response variables to elucidate linear dependencies, specifically by maximizing the covariance between the spectral signal and material composition. As shown in Equations 16–18, the mathematical decomposition for the spectral matrix and chemical composition values is expressed as

$$X=TP^{T}+E$$ (16)

$$Y=UQ^{T}+F$$ (17)

Y=XB+D (18)

where, $X\in R^{m\times n}$ represents the m×n spectral predictor matrix (comprising n samples and m wavelengths), and Y denotes the n×p response variable matrix. T , U signifies the score matrix representing the projection of the latent structure, P,Q describes the loading matrix indicating the orientation of the latent space. Furthermore, E , F refer to the residual matrices containing noise or unmodeled variance, with B , D representing the regression coefficients and the error term, respectively.

PLS remains one of the most widely used calibration methods in process spectroscopy because it can handle highly collinear spectral variables using a relatively small number of latent variables. Its low computational burden, transparent latent-variable structure, and straightforward model updating make it particularly suitable for applications with limited calibration samples, stable process conditions, and embedded computing constraints. However, PLS performance can degrade when the calibration domain is affected by linear scattering effects, temperature-induced peak shifts, or substantial process drift. In such cases, preprocessing, variable selection, local modeling, nonlinear regression, or adaptive updating should be evaluated before concluding that a more complex model is required.

In dynamic industrial environments, the transition from linear chemometrics to advanced deep learning architectures is not merely a computational trend, but a mechanistic necessity driven by severe nonlinear spectral distortions. These nonlinearities originate from specific, uncalibrated physical interferences. For instance, severe Mie scattering in highly heterogeneous matrices alters the effective optical pathlength in a wavelength-dependent, nonlinear manner. Furthermore, thermal fluctuations induce anharmonic shifts in hydrogen-bonded overtones (e.g., water bands at 1,450 nm and 1940 nm), while dynamic detector window contamination causes complex, wavelength-dependent baseline warping. Under such multi-stress industrial conditions, the relationship between spectral intensity and analyte concentration becomes highly nonlinear, fundamentally violating the additive assumptions of PLS. When forced to fit these nonlinearities, PLS often incorporates excessive latent variables, leading to severe overfitting and poor transferability across different process batches (Dong et al., 2025; Wold et al., 2001a; Zhu et al., 2023).

Although PLS offers high computational efficiency, its strict linear assumption limits its performance in complex matrices. The heterogeneity of samples such as tobacco, pulverized coal, and paper leads to non-linear spectral variations. Therefore, PLS often requires integration with rigorous variable selection algorithms, generally adopting Competitive Adaptive Reweighted Sampling (CARS), to manually eliminate uninformative bands.

Kernel-based methods such as SVR and LS-SVM provide nonlinear modeling capability without requiring the large training datasets typically associated with deep neural networks. They are often suitable for small-to medium-sized spectral datasets and can achieve competitive performance when kernel and regularization parameters are properly optimized. Their limitations include sensitivity to parameter selection, increasing computational cost with larger datasets, they represent an independent modeling option rather than merely a transitional stage (Huang et al., 2023; Sitorus and Lapcharoensuk, 2023). In complex chemical reactions involving pharmaceutical mixing and fermentation broths, necessitating the introduction of machine learning strategies. LS-SVM utilizes a non-linear kernel function to map input vectors into a high-dimensional feature space, constructing a linear decision surface to distinguish complex data. As shown in Equation 19, the prediction function derived from the optimization problem in LS-SVM is

$$y\left(x\right)=\sum_{k=1}^{N}\alpha_{k}K\left(x,x_{k}\right)+b,$$ (19)

where, $y\left(x\right)$ denotes the predicted value for the new spectral input, $\alpha_{k}$ represents the Lagrange multipliers obtained during the training phase. Furthermore, $K\left(x,x_{k}\right)$ serves as the kernel function used to quantify the similarity between the input and the training samples, and b corresponds to the bias term.

LS-SVM can exhibit superior performance over PLS in handling non-linear relationships under certain conditions (e.g., when sufficient and representative calibration data are available), this advantage is not universal. In practice, machine learning models such as LS-SVM require careful manual hyperparameter tuning (including regularization parameters and kernel width) and rely on feature wavelength selection to mitigate overfitting. Their effectiveness is highly contingent on data quality, sample size, and the degree of process nonlinearity. In many industrial scenarios, particularly those with limited calibration samples or relatively stable linear process behavior, PLS may remain a competitive or even preferable alternative. Consequently, the relative merits of these algorithms should be assessed on a case-by-case basis, rather than presuming the general superiority of any single approach. Comparative studies have shown that the performance gap between deep learning and PLS is often situational, and in some cases deep learning does not yield significant improvements. With the advancement of artificial intelligence and machine learning technologies, deep learning has begun to lead technological development. In the data analysis sector of online infrared spectroscopy, 1D-Convolutional Neural Networks (1D-CNN) have become the mainstream architecture. Advancing beyond traditional dimensionality reduction techniques that treat spectra as disordered vectors, the proposed method utilizes a 1D-CNN architecture to explicitly leverage the local continuity inherent in spectral data (Acquarelli et al., 2017; Peng et al., 2024; Yan, 2025). It realizes an adaptive learning process from input to output, extracting features from raw spectra without the need for manual feature spectral selection. As shown in Equation 20, its core operation is the 1D convolution, which involves the sliding of a kernel function over the spectral sequence

$$s\left(t\right)=\left(x*\omega \right)\left(t\right)=\sum_{a=0}^{k-1}x\left(t-a\right)\omega \left(a\right)$$ (20)

where, $s\left(t\right)$ denotes the feature map output at position t , and x represents the input spectral intensity sequence. Additionally, ω signifies the learnable weight vector of length k , while a corresponds to the relative position within the convolution window.

1D-CNNs are well suited to spectral data. Their convolutional kernels capture local band shapes and hierarchical feature interactions. With sufficiently large and representative training datasets, they can improve modeling of complex nonlinear relationships and reduce reliance on manually selected wavelengths. These advantages are not universal. When the dataset is small, class distributions are imbalanced, production batches are insufficiently represented, or external validation is absent, 1D-CNNs may not outperform simpler methods. Pooling and convolution offer limited tolerance to spectral shifts, but they do not eliminate temperature drift, sensor aging, calibration transfer problems, or extrapolation beyond the training domain. Thus, 1D-CNNs should be chosen only when the expected benefit is supported by adequate data, independent-batch validation, and sufficient computational resources. They should be benchmarked against optimized PLS and lightweight nonlinear models, not assumed superior *a priori*. Within strongly nonlinear regimes, properly designed 1D-CNNs offer clear advantages. Their local kernels act as adaptive filters that capture localized spectral shapes independent of baseline wandering, providing translation invariance against peak shifts. Nonlinear activations (e.g., ReLU) enable modeling of coupled scattering-absorption interactions that linear models cannot resolve. Consequently, 1D-CNNs do not replace PLS in controlled environments, but serve as a useful soft-calibration framework when optical interferences exceed the capacity of traditional preprocessing (Gamagedara et al., 2025).

As the number of layers in a CNN network increases, features become increasingly abstract; shallow convolutional layers capture local gradients, whereas deep convolutional layers abstract global spectral signatures (functional group fingerprints). Recent studies on tobacco drying have shown that, under certain conditions, a 1D-CNN can outperform CARS-PLS by automatically suppressing noise and focusing on moisture-relevant bands (Deng et al., 2026). However, such improvements are not guaranteed; they depend critically on dataset size, diversity, and the degree of nonlinearity. In many industrial scenarios with limited calibration samples or stable linear processes, PLS may still offer comparable or even better predictive performance.

The evolution of chemometric algorithms in process analytical technology is fundamentally driven by the necessity to digitally compensate for the three major classes of interferences, optical, physical, and thermodynamic that optical hardware cannot fully overcome. Table 2 represent the advantages and disadvantages of the algorithm.

**TABLE 2:** Comparison of chemometric and deep learning models under industrial online interferences.

Model	Nonlinear capability	Online updating	Performance under peak shift	Feature extraction mechanism	Industrial online suitability	Main limitations
PLS (Shaikh and Uzgare, 2026)	Limited	Relatively easy	Fails; cannot handle unaligned peaks	Global dimension reduction (PCA-like)	Requires strictly controlled matrices	Sensitive to unmodeled nonlinearity and domain drift
LS-SVM (Balabin and Lomakina, 2011)	Moderate–high	Moderate-cost	Moderate; mitigated by Kernel functions	Manual preprocessing + Kernel mapping	Struggles with extreme multi-stress	Parameter sensitivity and scaling cost
1D-CNN (Cui and Fearn, 2018)	High	Possible but costly	Excellent; achieved via pooling (Translation Invariance)	Automated local hierarchical filtering	Ideal for dynamic, multi-stress environments	Overfitting, data leakage, limited transferability

The values in the table are provided as engineering guidance and should be validated for each specific spectrometer, optical configuration, and industrial operating condition.

PLS regression, strictly founded on linear assumptions, exhibits exceptionally poor robustness against coupled multi-stress interferences. When subjected to severe optical baseline warping and physical Mie scattering, PLS erroneously integrates excessive latent variables to fit macroscopic noise. Quantitative studies indicate that under such non-linear distortions, the predictive coefficient of determination ( $R^{2}$ ) for PLS often rapidly deteriorates below 0.80, with the Root Mean Square Error of Prediction (RMSEP) increasing by 200%–300% (Shaikh and Uzgare, 2026). This completely masks minute target variances (e.g., strict moisture fluctuations within a 7.16%–7.19% margin), causing catastrophic model failure. In more controlled or linear regimes, PLS remains a reliable and efficient tool.

To address shallow nonlinearities, Least Squares Support Vector Machines (LS-SVM) offer a transitional solution with moderate interference resistance. By utilizing kernel functions (e.g., RBF) to map data into high-dimensional linear spaces, LS-SVM establishes non-linear decision boundaries. Compared to PLS, LS-SVM can reduce RMSEP by 15%–20% when handling moderate optical attenuation and physical scattering. However, its effectiveness remains heavily dependent on rigorous manual preprocessing (e.g., SNV/MSC) (Balabin and Lomakina, 2011). Furthermore, under extreme dynamic conditions, it struggles to autonomously compensate for thermodynamic peak shifts, limiting its robustness.

In contrast, 1D Convolutional Neural Networks (1D-CNN) demonstrate profound resistance across all three interference categories. Mechanistically, 1D-CNNs eliminate reliance on global baselines. Their localized convolutional kernels act as adaptive filters that inherently ignore optical baseline drifts and physical scattering distortions. Simultaneously, the network’s pooling layers provide translation invariance, effortlessly tolerating thermodynamic peak shifts and spectral jitter. Quantitative comparisons reveal that in extreme multi-stress environments, 1D-CNNs can maintain an $R^{2}>0.95$ and reduce RMSEP by over 30%–50% relative to PLS (Cui and Fearn, 2018). Nevertheless, these gains are conditional; they require large, well-annotated datasets and are not consistently observed across all industrial applications. In fact, several investigations have found that when the training set is small or the process is largely linear, deep learning offers no significant advantage over PLS (see, e.g. (Banerjee et al., 2024)). Therefore, the choice of calibration algorithm should always be guided by the specific characteristics of the process and the available data, rather than by an *a priori* preference for any single method.

In industrial online spectroscopy, model degradation arises not only from limited nonlinear capacity but also from time-varying data distributions caused by raw-material variation, sensor aging, probe fouling, instrument replacement, and operating-condition changes, all of which induce concept drift or calibration-domain shift. Replacing a conventional model with a more complex static one does not necessarily resolve these issues. Maintenance strategies such as recursive PLS, moving-window calibration, incremental learning, locally weighted modeling, calibration transfer, and periodic recalibration using fresh samples can sustain performance. The choice depends on drift rate, reference data availability, and maintenance burden. Ultimately, no single algorithm is universally optimal: PLS suits small datasets and applications valuing interpretability and efficiency; LS-SVM handles moderate nonlinearity with modest data; 1D-CNN fits large-scale, complex nonlinear problems given sufficient data and rigorous validation. These approaches are complementary, not a simple linear progression, and their applicability is governed by process conditions, spectral characteristics, and deployment constraints.

### 3.3 Guidelines and pitfalls for deep learning in process spectroscopy

While deep learning offers unprecedented nonlinear modeling capabilities, its application in vibrational spectroscopy is fraught with methodological pitfalls that frequently lead to catastrophic model failure in industrial settings. A primary issue is the uncritical adoption of network architectures. Unlike high-resolution 2D image processing, analyzing 1D spectral vectors (typically containing 1,000 to 2,000 collinear variables) does not require exceptionally deep architectures like ResNet-152 or VGG-16. Deploying such ultra-deep networks—often containing millions of trainable parameters—on relatively small spectral datasets inevitably triggers the Hughes phenomenon (curse of dimensionality), causing the network to memorize high-frequency instrument noise and transient baseline fluctuations rather than underlying chemical absorbance. Empirical evidence suggests that for process spectroscopy, a physically informed, shallow 1D Convolutional Neural Network (1D-CNN) is vastly superior. Optimal architectures typically comprise only 2 to 4 convolutional layers with relatively large kernel sizes (e.g., 5 to 15 data points) to effectively capture broad vibrational overtones, followed by max-pooling layers. This structural constraint reduces the trainable parameter space to merely 10^4^ magnitude. Coupled with aggressive regularization techniques—such as applying Dropout rates of 0.3–0.5 and implementing Early Stopping based on an independent validation loss plateau—these shallow networks can robustly extract chemical variance without overfitting to the calibration set (Balabin and Lomakina, 2011; Cui and Fearn, 2018).

Furthermore, the most pervasive and statistically fatal error in industrial spectral modeling is the phenomenon of data leakage induced by uncritical sampling strategies. In continuous-flow or batch manufacturing processes (such as dynamic twin-screw extrusion or powder blending), spectral data exhibit strong temporal autocorrelation. Adjacent samples acquired at high frequencies share nearly identical physicochemical states and physical scattering profiles. For instance, if a dataset contains 5,000 spectra collected across 5 distinct production batches, applying standard randomized k -fold cross-validation will inevitably distribute highly correlated, temporally adjacent spectra into both the training and testing sets. Consequently, the network “predicts” the test set by simply recognizing the unique baseline signature of that specific time-point, yielding a deceptive illusion of near-perfect accuracy (frequently reporting $R^{2}>0.99$ and artificially deflated Root Mean Square Errors). However, when this overfitted model is transferred to a completely new, independent manufacturing batch, this artificially inflated performance collapses immediately, with prediction errors often deteriorating by an order of magnitude.

To conclusively ensure genuine predictive robustness, practitioners must abandon randomized cross-validation and employ validation protocols that rigorously respect the macroscopic process dynamics. Specifically, chronological data splitting (e.g., training a model on spectra from Days 1–3 and strictly testing it on unseen data from Day 4) and Leave-One-Batch-Out (LOBO) cross-validation are imperative. By intentionally forcing the 1D-CNN to predict a completely independent, temporally isolated process trajectory, LOBO correctly penalizes models that overfit to batch-specific environmental noise or uncalibrated sensor drift (Cheveralls et al., 2026). Deep learning can become a reliable Process Analytical Technology (PAT) tool only under strict, physically constrained validation frameworks (Kaufman et al., 2012). Deep learning models offer powerful nonlinear modeling capability. They should not, however, be viewed as a universal substitute for traditional chemometric methods. Model selection depends on data characteristics, computational resources, and application needs. PLS and LS-SVM remain preferable for small datasets and cases where interpretability and computational efficiency are critical. 1D-CNN is better suited to large-scale datasets with complex nonlinear spectral relationships. Choosing the right modeling strategy for each industrial scenario is therefore essential to achieve robust and reliable spectroscopic analysis.

### 3.4 Industrial lifecycle management for online spectroscopic calibration

Deploying online spectroscopic models in continuous industrial processes requires balancing predictive capacity with practical operational realities. While academic benchmarks emphasize offline validation error, field deployment subjects calibration algorithms to severe physical constraints, including optical source degradation, sensor window fouling, ambient temperature swings, raw material lot fluctuations, and limited edge-hardware compute capacity. Consequently, algorithm selection cannot rely solely on reported accuracy under static conditions; it must evaluate how base modeling paradigms, PLS, LS-SVM, and 1D-CNN, interact with adaptive online maintenance strategies over the long-term instrument lifecycle.

Each baseline modeling framework presents distinct operational advantages and vulnerabilities when exposed to industrial dynamics. PLS remains the industrial workhorse due to its minimal computational footprint, low training sample requirements, and complete transparency through latent variable structures. In field settings, PLS enables real-time execution ( τ<1ms ) on low-power programmable logic controllers (PLCs) and allows process engineers to directly trace spectral load vectors to physical functional groups. However, PLS fails when non-linear physical phenomena, such as light scattering variations in high-solid slurries or severe optical pathlength changes, dominate the system, resulting in elevated prediction bias ( $e=y-\hat{y}$ ). LS-SVM addresses these non-linear relationships through kernel mapping without requiring massive datasets, making it effective for moderately complex chemical matrices. Its chief operational limitation lies in its black-box nature, heavy reliance on precise hyperparameter tuning $\left(\gamma ,\sigma^{2}\right)$ , and high computational memory scaling during inference, which restricts its execution on resource-constrained embedded edge devices.1D-CNN eliminates manual feature selection by automatically learning spatial spectral representations, achieving unmatched accuracy in highly non-linear, multi-component systems. Nevertheless, its industrial implementation is heavily constrained by the necessity of massive calibration sets ( N>1000 ) to prevent catastrophic overfitting, high power consumption requiring dedicated GPU accelerators, and an extreme vulnerability to baseline drift, where minor spectral shifts can cause silent model failure across complex feature-extraction layers (Chauhan et al., 2019; Li et al., 2021; Wold et al., 2001b).

To prevent longitudinal performance degradation without incurring the prohibitive cost of frequent laboratory assays, these base models must be mathematically integrated with online adaptive wrappers: Calibration Transfer (CT), Recursive Partial Least Squares (RPLS), and Moving Window or Just-In-Time (JIT) local adaptation. Each adaptive mechanism offers specific field advantages while introducing distinct operational trade-offs (Rinnan et al., 2014; Urhan and Alakent, 2020; Workman, 2018).

Calibration transfer using Piecewise Direct Standardization (PDS) solves instrument replacement and sensor swapping challenges by mapping target instrument spectra $X_{target}$ to a master unit matrix using a localized linear transformation $X_{std}=X_{target}F$ (Chen et al., 2022). Industrially, PDS allows a single master calibration model to be deployed across an entire factory network without re-analyzing hundreds of standard reference samples. When integrated with PLS, the transformation matrix F acts as a lightweight pre-processing layer, maintaining real-time execution speeds ( <2ms ). For LS-SVM and 1D-CNN, PDS prevents expensive full-model retraining by standardizing input features before kernel or convolutional operations. However, PDS requires maintaining physical transfer standards, and its performance degrades if non-linear optical distortion differs between instruments.

For continuous, slow-varying process drift, such as optical lamp aging or gradual window dust accumulation, recursive parameter updating provides a seamless maintenance framework. Recursive PLS (RPLS) integrates directly into the PLS algorithm by updating the cross-covariance vector $S_{t}=\lambda S_{t-1}+y_{t}X_{t}$ upon receiving a new reference assay $\left(X_{t},y_{t}\right)$ , where $\lambda \in (\left.0,1\right]$ represents an exponential forgetting factor. The primary field advantage of RPLS is its ability to automatically track steady baseline drift without stopping production or altering model architecture (Rinnan et al., 2014). When applied to non-linear models, incremental learning updates the dual weight parameters of LS-SVM or fine-tunes only the final fully connected layers of a 1D-CNN using low learning rates. While highly effective for steady decay, recursive strategies suffer from a key field drawback: if an erroneous laboratory reference assay is ingested, the updating step permanently corrupts the model parameters until sufficient correct data arrives.

When processes experience abrupt operational shifts, non-monotonic product grade changes, or cyclical matrix variations, Moving Window (MW) and Just-In-Time (JIT) localized modeling strategies are necessary. Moving Window PLS (MWPLS) discards outdated historical spectra outside a fixed sliding temporal window W , continually re-estimating model parameters on the most recent process state. JIT modeling goes further by bypassing global model training entirely; for every new online spectrum $X_{query}$ , JIT computes Mahalanobis distances $d\left(X_{i},X_{query}\right)$ across a historical database, selects the k− most similar historical spectra, and constructs a dynamic, localized model on the fly (Zheng et al., 2021). JIT-PLS and JIT-LS-SVM handle severe nonlinearities and sudden feedstock changes well. Local subspaces remain approximately linear. For 1D-CNNs, windowed adaptation uses continual learning with small experience-replay buffers. The main drawback of JIT and windowed strategies is storage and computational cost. Searching large historical databases and fitting local models for each scan increases latency. This makes them unsuitable for sub-second closed-loop control on low-cost edge controllers.

Sustainable deployment requires matching the base model and adaptive strategy to the process drift and hardware resources. For stable, linear processes on embedded microcontrollers, PLS with RPLS or PDS works well. It delivers low-latency, self-maintaining predictions. For complex, nonlinear systems with abundant historical data and dedicated edge computing, 1D-CNN or LS-SVM are justified. They must use JIT local adaptation or periodic transfer standardization to ensure long-term stability. Table 3 summarizes both base models (PLS, LS-SVM, 1D-CNN) and adaptive strategies (PDS, RPLS, JIT/MW) against key operational metrics: computational latency ( Δτ ), sample demand ( N ), field limitations, and deployment integration mechanisms.

**TABLE 3:** Comparative evaluation of spectroscopy modeling and adaptive calibration strategies.

Method/Strategy	Industrial advantage	Field limitation	Integration with PLS/LS-SVM/1D-CNN	Latency impact ( Δτ )	Sample Efficiency ( $N_{ref}$ )	Typical scenario
PDS (Chen et al., 2022)	Transfer error reduced by>80%	Fails if non-linear optical error >15%	Linear filter: $X_{std}=X_{target}F$	<1 ms	5-15std. Samples	Sensor/lamp swap
RPLS (Rinnan et al., 2014)	Tracks baseline drift up to 2% month	1 outlier assay corrupts weight matrix	Dynamic update: $S_{t}=\lambda S_{t-1}+y_{t}X_{t}$	<2 ms	1 assay/step	Aging, dust fouling
JIT/MW (Zheng et al., 2021)	Grade shift RMSEP within ±5%	RAM >500 MB Search: $(O\left(N\cdot P\right)$	Dynamic local fit on k− nearest spectra	10–50 ms	>500 DB samples	Grade/feedstock shift
PLS	Ultra-low compute; linear transparency	Fails under severe physical non-linearities	Base linear regressor y=Xβ	<1 ms	30–100	Linear processes on basic PLCs
LS-SVM	Fits non-linear matrix with small N	Black-box; memory scales as $O\left(N^{2}\right)$	Non-linear kernel mapping $K\left(X_{i},X^{*}\right)$	5–15 ms	100–300	Moderate non-linear reactions
1D-CNN	Auto spatial features; no λ -selection	High data demand; risk of overfitting	End-to-end spatial convolution	>50 ms	>1,000	Complex matrix with edge GPUs

## 4 Application strategies and development trends of online measurement infrared monitoring technology

**FIGURE 10:**
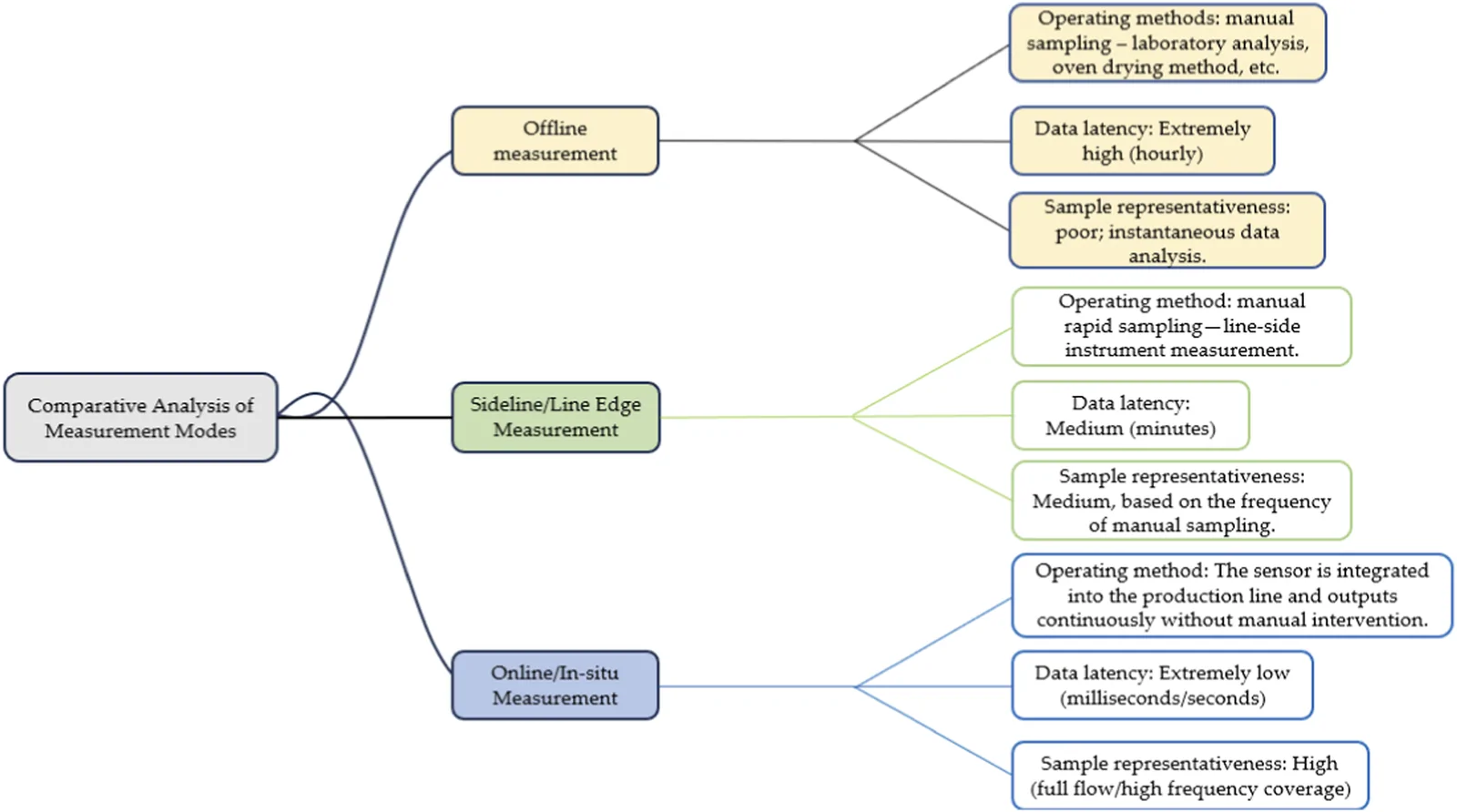
Technology roadmap for different measurement modes.

Process analysis technology is primarily categorized into three methodological approaches: offline, bypass, and online measurement. Offline and bypass configurations are constrained by their inability to capture temporal variations in real-time, thereby precluding the early detection of process deviations. In contrast, online measurement entails the direct integration of detection instrumentation onto processing vessels or conveyor systems. This facilitates the continuous, automated, and real-time characterization of material states and composition, enabling immediate data feedback to the process control system. Characterized by real-time capability, non-destructiveness, *in situ* operation, and high throughput, online process analysis provides essential support ranging from closed-loop feedback during production to quality verification upon product completion. Notably, online Near-Infrared spectroscopy, distinguished by its non-invasive nature, non-destructive analysis, and instantaneous multi-component detection, has emerged as a critical methodology for achieving refined control and adaptive regulation in industrial production. It currently represents the most mature and rapidly expanding technique within the domain of online measurement (Gao et al., 2014; Klozinski and Barczewski, 2019; Semyalo et al., 2024). Figure 10 illustrates the technical roadmap characteristics of different measurement modes.

**FIGURE 11:**
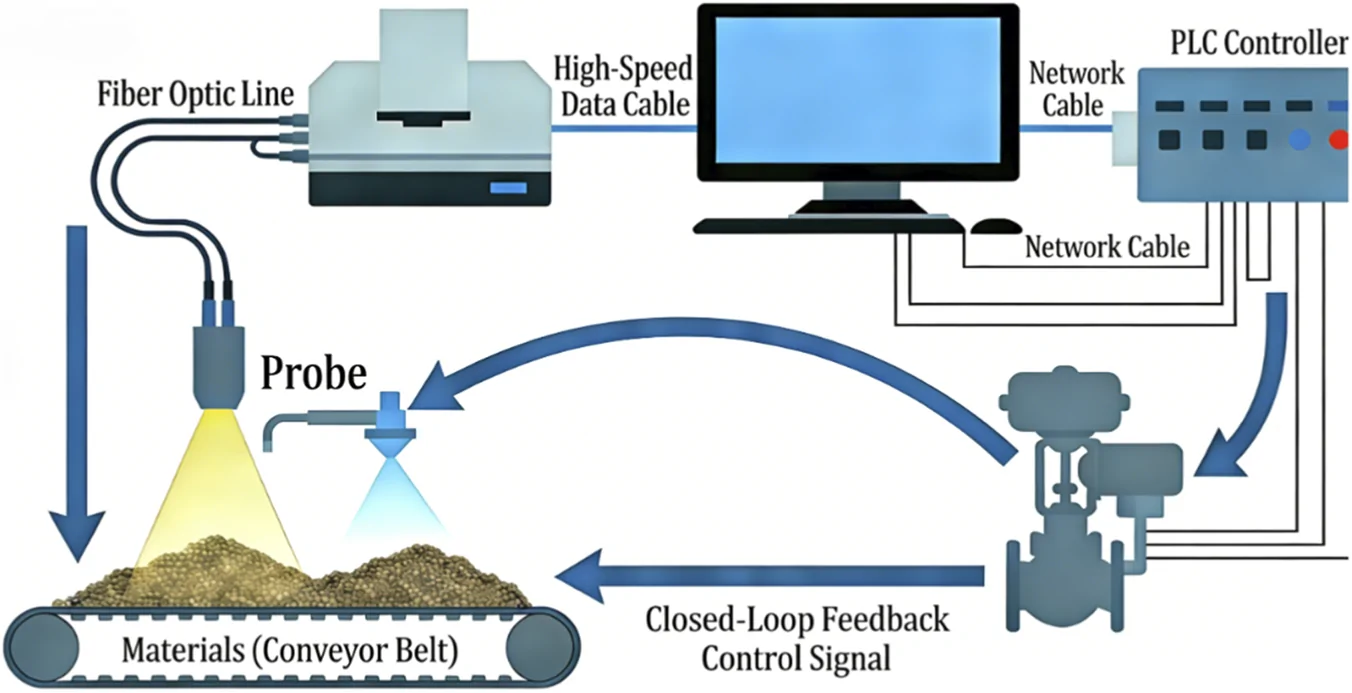
Online spectroscopic detection system structure diagram.

The application strategy for IR analyzers must balance external factors with internal optical architectures, seeking an optimal equilibrium across diverse instrumental principles regarding material types and measurement ranges. Given that industrial environmental factors such as temperature, humidity, and dust, alongside material physical states, significantly impact online measurement and introduce errors in data stability, it is imperative to mitigate external interferences (Sun et al., 2020). Furthermore, careful consideration must be given to the specific application scopes of Filter-type, Fourier Transform, Grating dispersion, and Acousto-Optic Tunable Filter technologies, as well as algorithmic optimization and system integration. Currently, numerous research groups worldwide are conducting in-depth investigations into the integrated development of NIR spectroscopy. Research spans from fixed-wavelength index monitoring and rapid random-access wavelength closed-loop control to high-precision complex component analysis and high-speed monitoring of non-homogeneous materials. These efforts aim to adapt to diverse production scenarios, material states, and environmental conditions, leading to the development of specialized online NIR instrumentation (Workman, 2018). Regarding the selection of infrared spectroscopic technologies, extensive literature and research initiatives have systematically evaluated various instrument types. These studies analyze typical industries, analytical states, core advantages, and limitations. To accommodate varying on-site environments, material characteristics, and industry-specific requirements, instrument adaptation must factor in resolution, response time, and measurement threshold intervals (Levine et al., 1989; Mayr et al., 2021; Shi and Yu, 2017). Figure 11 displays the structural diagram of the online spectral detection system.

**FIGURE 12:**
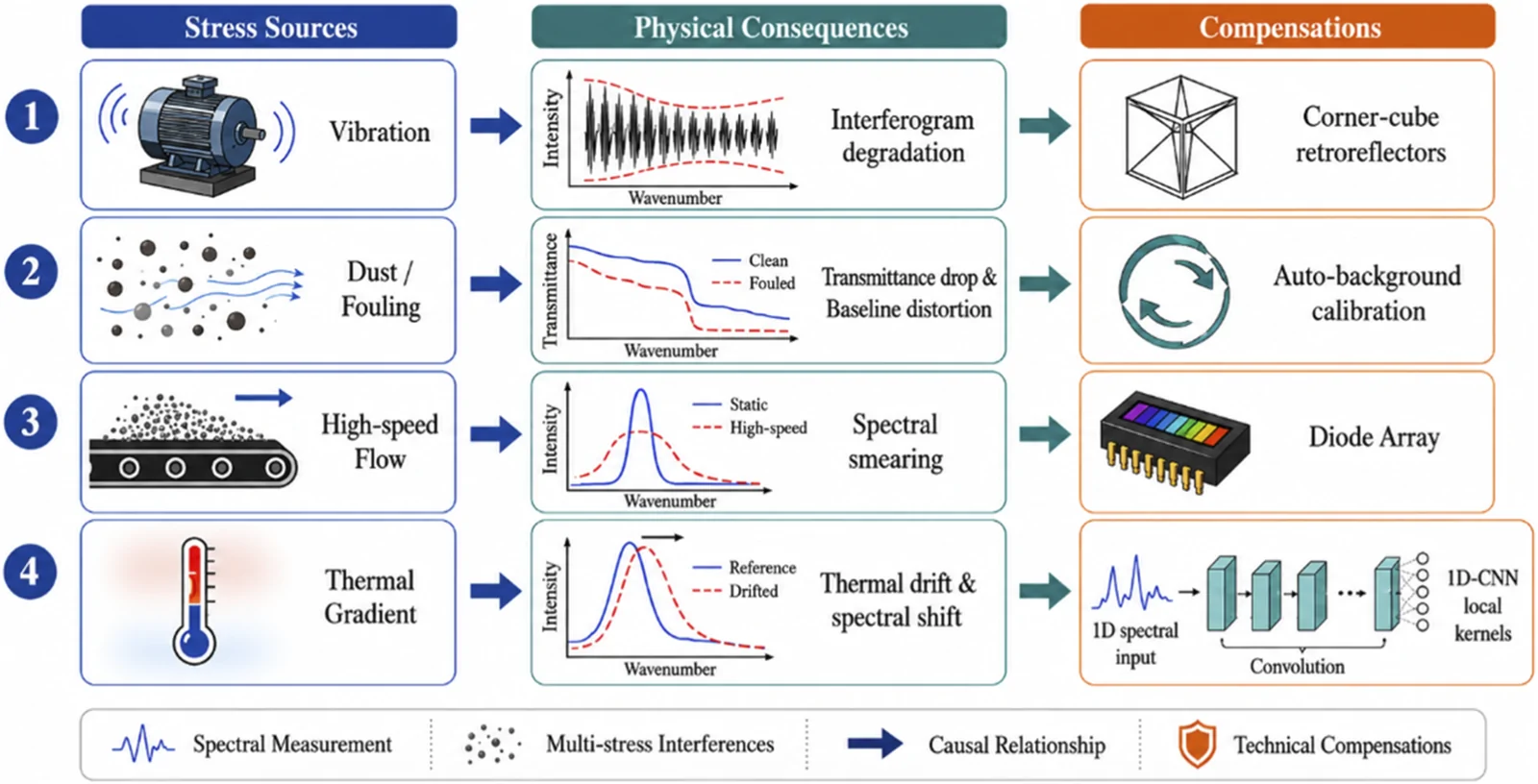
Mechanisms of multi-stress interferences and technical compensations.

In online spectral measurement, it is crucial to clearly delineate the “coupled multi-stress interferences” inherent to online spectroscopic measurements. These interferences, which fundamentally compromise predictive accuracy, can be classified into three primary categories. First, optical and hardware interferences mainly include drastic reductions in the effective optical aperture due to window pollution, as well as non-linear attenuation of optical throughput and severe baseline drift caused by light source fluctuations. Second, physical state interferences involve dynamic Mie scattering induced by heterogeneous particle sizes and varying bulk densities (which severely distorts absorption spectral morphology), alongside high-speed spatiotemporal misalignment caused by fast-moving materials. Third, thermodynamic interferences arise from drastic ambient temperature fluctuations, which not only trigger optical distortions within instrument components but also alter the internal hydrogen-bonding networks of the matrix, leading to anharmonic shifts in characteristic absorption peaks (Batten, 1998). Figure 12 is represent Mechanisms of Multi-stress Interferences and Technical Compensations.

Although filter-based, grating-based, Fourier-transform, and AOTF spectrometers differ substantially in wavelength selection and signal acquisition, their online performance is affected by a common set of industrial disturbances, including probe-window fouling, source-intensity fluctuations, detector drift, temperature variation, mechanical vibration, changes in particle size and surface morphology, fluctuations in material distance or layer thickness, and the rapid motion of heterogeneous samples (Scheibelhofer et al., 2014). The manifestation of these disturbances, however, is strongly dependent on the optical architecture of the instrument. Filter-based spectrometers are primarily constrained by limited spectral redundancy and channel-response drift, making it difficult to distinguish sample variation from contamination-induced attenuation. Grating-based systems are more susceptible to wavelength-dependent scattering, array-response nonuniformity, full-spectrum baseline distortion, and prediction outside the calibration domain. In Fourier-transform spectrometers, contamination and vibration first degrade the interferogram, leading to reduced centre-burst amplitude, phase instability, background mismatch, and pseudo-absorption features. AOTF spectrometers, by contrast, are mainly affected by the coupled influence of crystal temperature, radio-frequency stability, wavelength-dependent diffraction efficiency, and sequential-scanning mismatch (Martens and Stark, 1991). Accordingly, interference control for online spectroscopy should not rely on a single universal correction method, but should be tailored to the dominant failure mechanism of each spectrometer architecture.

### 4.1 Channel interferences and correction in online filter-based spectroscopy

Filter-based spectrometers generally monitor only a limited number of preselected wavelengths, typically including one or several analyte-sensitive bands together with a reference band. In online moisture measurement, the reference channel is intended to compensate for variations in source intensity, optical throughput and sample presentation. This compensation is effective only when the analytical and reference channels are attenuated to a similar extent. Under industrial conditions, however, deposited dust rarely behaves as a spectrally neutral layer. Differences in particle size, colour, refractive index, moisture content and chemical composition can produce wavelength-dependent scattering and absorption. As a result, the analytical and reference channels may no longer decline proportionally, and the channel ratio may change even when the actual moisture content remains unchanged. For example, if the analytical channel retains 80% of its clean-window signal while the reference channel retains 90%, the resulting ratio deviates by approximately 11.1% from the clean condition, despite an average optical loss of only 15%. This demonstrates that differential channel attenuation, rather than overall signal loss alone, is the principal contamination risk for filter-based instruments.

The limited number of wavelength channels also restricts the ability of filter-based systems to distinguish process variation from instrumental disturbance. If deposited water, oil or organic residues absorb near the selected moisture-sensitive wavelength, the resulting channel-ratio change may be incorrectly interpreted as a genuine increase or decrease in material moisture (Juntunen, 2025). Unlike continuous-spectrum instruments, filter-based systems cannot readily use spectral-shape changes, Q residuals or multivariate distance statistics to identify such interference. Their diagnostic capability must therefore rely on the temporal consistency of individual channels, the stability of reference signals and the use of additional monitoring channels. In practical implementation, a three-channel configuration comprising an analytical channel, a constituent-insensitive reference channel and a fouling-monitoring channel is preferable to a simple dual-wavelength design. The fouling-monitoring wavelength should be located outside the principal absorption region of the target constituent so that it primarily reflects changes in window transmittance (Bekaert et al., 2022).

In addition to probe fouling, filter-based online instruments are affected by source ageing, detector gain drift, temperature variation, sample-distance fluctuations, material-layer thickness, particle-size variation, surface roughness and ambient-light leakage (Claßen et al., 2017). Source ageing generally causes a gradual decrease in both the sample and reference signals, whereas window contamination mainly affects the sample-facing optical path. An independent reference detector positioned before the process window can therefore help distinguish lamp degradation from optical-path contamination. If both the sample and reference detectors decline at a similar rate, source ageing is more likely; if the reference detector remains stable while the sample channels decrease, window fouling or optical obstruction is the more probable cause. Detector drift should also be considered because a gain change of only 1%–2% may be comparable to the signal variation associated with a small moisture change in narrow-range industrial applications (Lazaro-Pacheco et al., 2026).

A graded intervention strategy is more appropriate than unrestricted ratio correction. As an initial engineering scheme, a reduction of less than 10% in reference-channel intensity relative to the clean-window condition may be regarded as mild attenuation, provided that channel imbalance remains small and the prediction error remains within the validated RMSEP. A decrease of 10%–20% should trigger more frequent reference measurements, shortened background-update intervals and pulsed air purging. When the retained signal falls below approximately 70%–80% of the clean-window level, or when the analytical and reference channels exhibit clearly unequal attenuation, dual-wavelength compensation should no longer be considered reliable. Window cleaning, temporary suppression of the reported value or transfer to a redundant sensor is then required. These ranges should be treated as provisional engineering limits and validated for the specific wavelength combination, detector dynamic range, optical geometry and required measurement accuracy (Asachi and Camargo-Valero, 2023; Mattarozzi et al., 2023).

Because filter-based instruments provide limited spectral redundancy, hardware protection is generally more important than complex post-processing. Air curtains and positive-pressure purging can reduce particle deposition, while inclined or flush-mounted windows can decrease direct particle impact. Pulsed purging is particularly useful when continuous airflow would disturb the material layer, change bulk density or introduce additional measurement variability. Window heating may be applied under condensation-prone conditions, although temperature stability must be maintained to avoid introducing detector drift or changing the optical properties of the process material. A reliable protection strategy should therefore combine physical fouling prevention, independent source monitoring, multiple optical channels, threshold-based cleaning and automatic suppression of invalid outputs. Algorithmic compensation should be used only within a validated operating region in which the signal-to-noise ratio remains sufficient and the channel response remains reversible. Table 4 is represent main online interferences and Corrective measures for filter-Based spectrometers.

**TABLE 4:** Main online interferences and corrective measures for filter-based spectrometers.

Interference source	Quantitative manifestation	Main diagnostic evidence	Recommended corrective measure	Suggested intervention level
Uniform window attenuation	Analytical and reference channels decline at similar rates	Comparable reduction across monitored channels	Ratio correction and shorter reference interval	Reference-channel loss below approximately 10%
Wavelength-selective fouling	Analytical and reference channels decline unequally	Channel imbalance and progressive ratio drift	Add a fouling-monitoring wavelength, recalibrate or clean the window	Channel difference of approximately 2%–5% as warning; above 5% as intervention
Severe window fouling	Large reduction in signal intensity and SNR	Reference-channel retention and repeatability	Air purge, mechanical cleaning or output inhibition	Retained signal below approximately 70%–80%
Source ageing	Sample and source-reference signals decline simultaneously	Stable sample/reference ratio despite absolute signal loss	Source normalization or lamp replacement	Drift outside the validated source-reference band
Detector gain or temperature drift	Slow offset or gain change without optical-path change	Detector temperature, dark signal and reference signal	Temperature compensation and periodic dark correction	Gain drift above approximately 1%–2% in high-precision applications
Sample-distance or layer-thickness fluctuation	Rapid common-mode intensity variation and poor repeatability	Increased short-term coefficient of variation	Fix probe distance, increase optical footprint and apply temporal averaging	CV above the process-specific repeatability limit
Particle-size or surface-scattering variation	Channel ratio changes without a true moisture change	Inconsistency among multiple reference channels	Include particle-size variation in calibration	Prediction error above the validated RMSEP
Ambient-light leakage	Additive offset, particularly at low source intensity	Difference between source-on and source-off measurements	Optical shielding and synchronous detection	Background contribution above approximately 1%–2% of the useful signal
Condensation or oil-film formation	Nonlinear and wavelength-selective attenuation	Persistent channel imbalance and abnormal temperature or visual diagnosis	Window heating, dry purge and cleaning	Channel imbalance persists after background update
Model invalidation	Apparently stable prediction despite abnormal raw signals	Prediction bias, RMSEP and validity flag	Suppress output and request maintenance	Prediction bias above approximately twice the RMSEP

The values in the table are provided as engineering guidance and should be validated for each specific spectrometer, optical configuration, and industrial operating condition.

### 4.2 Spectral distortion and anomaly detection in online grating-based spectroscopy

Grating-based spectrometers use linear or area detector arrays to acquire continuous spectra, providing rich information for monitoring optical contamination in industrial online applications, such as fluidized-bed NIR, powder processing, and biomass handling. However, several interference modes critically affect measurement reliability (Núñez et al., 2020).

Firstly, window fouling is the primary issue, reducing integrated spectral energy by 15%–25% and lowering SNR by ∼20% in fluidized-bed systems after several hours of operation. Preprocessing methods like SNV or MSC can restore apparent spectral shape, but cannot recover lost photon-limited chemical information. Energy retention below 10% is mild, 10%–20% is moderate, and >20–30% is severe, requiring background reacquisition, pulsed air purge, or window cleaning (Bogomolov et al., 2018; Zhang et al., 2025). Secondly, wavelength-dependent scattering arises from dust or particles 10–50 µm in size, producing baseline tilt of 0.5%–1.0% per 100 nm and curvature up to 1.5%–2.0% for thick deposits. Constant background subtraction cannot correct this nonuniform attenuation; SNV, MSC, EMSC, or detrending is required for mild cases, and physical cleaning for severe fouling (Wrobel et al., 2020). Thirdly, detector-array nonuniformity can cause localized spikes or striping. Pixel deviations of 1%–2% over critical spectral regions have led to prediction errors exceeding RMSEP, highlighting the need for periodic dark calibration, pixel-response correction, and automatic bad-pixel detection (Parra et al., 2014). Fourthly, high-speed or heterogeneous material motion introduces spectral averaging artifacts. Short-term spectral variance increased by 20%–30% in biomass conveyor experiments, causing apparent baseline fluctuations. Reducing integration time, averaging multiple rapid acquisitions, enlarging the optical footprint, and synchronizing acquisition with particle flow mitigates these errors (De Man et al., 2023). Fifthly, model extrapolation occurs when spectra fall outside the calibration domain due to cumulative fouling, particle-size changes, or environmental drift. In fluidized-bed NIR, 12% of spectra exceeded the 95% Q-residual control limit after 6 h. Predictions without validity checks (Q residuals, Hotelling T^2^, leverage) risk being unreliable (Cunha et al., 2026).

Practical mitigation integrates hardware and software. Short pulsed air cleaning (e.g., 4 s after each acquisition) reduced deposition by 15%–25%, preserving baseline stability and prediction accuracy (Semyalo et al., 2024). Probe design (sapphire windows, inclined or flush mounting, positive-pressure housing, mechanical wipers) further minimizes fouling. Light contamination may be corrected when integrated energy remains >90%, SNR >85–90%, and multivariate residuals within control limits. Moderate fouling (10%–20% energy loss, baseline slope change, or correlation coefficient <0.995) triggers background reacquisition and pulsed cleaning. Severe fouling (>20–30% energy loss, SNR <60–70%, spectral correlation <0.980, or Q residuals >99%) requires prediction suspension. Table 5 is represent Main Online Interferences and Corrective Measures for Grating-Based Spectrometers.

**TABLE 5:** Main online interferences and corrective measures for grating-based spectrometers.

Interference source	Quantitative indicator	Diagnostic evidence	Corrective measures	Suggested intervention
Window fouling	Integrated energy loss 15%–25%; SNR drop ∼20%	Total spectral energy, SNR	Background reacquisition, pulsed air purge, window cleaning	Mild <10%; Moderate 10%–20%; Severe >20–30%
Wavelength-dependent scattering	Baseline tilt 0.5%–1.0%/100 nm; curvature 1.5%–2.0%	Baseline slope/curvature	SNV/MSC/EMSC for mild; window cleaning for severe	5%–15% slope change moderate; >15% severe
Detector-array nonuniformity	Pixel deviation 1%–2%	Spikes, striping	Pixel response correction, dark calibration, bad-pixel replacement	Key pixel deviation >1–2%
High-speed particle motion	Short-term spectral variance increase 20%–30%	Consecutive spectral CV	Shorten integration, average multiple acquisitions, synchronize with particle flow	Variance >20–30% baseline
Model extrapolation	Q residual >95% CI	Q residuals, T^2^, leverage	Suppress prediction, update calibration domain	95% limit warning; 99% limit reject

The values in the table are provided as engineering guidance and should be validated for each specific spectrometer, optical configuration, and industrial operating condition.

### 4.3 Interferogram disturbance and stabilization in online fourier-transform spectroscopy

In Fourier-transform spectrometers, disturbances introduced during industrial online measurement first affect the raw interferogram and are subsequently propagated into the reconstructed spectrum. This behaviour differs from that of dispersive instruments, in which interference is usually observed directly as a change in individual wavelength channels. Deposits on the process window reduce the broadband radiant flux reaching the detector and consequently lower the centre-burst amplitude near zero optical path difference. Non-uniform contamination may further reduce fringe modulation, alter the interferogram envelope and increase the uncertainty associated with phase correction. After Fourier transformation, these changes appear as reduced signal-to-noise ratio, baseline curvature, pseudo-absorption bands and unstable quantitative predictions (Wessel et al., 2009). Although FT-NIR systems can provide high optical throughput and, in some commercial configurations, signal-to-noise ratios exceeding 20,000:1 under favorable conditions, this initial performance margin cannot compensate indefinitely for progressive contamination or loss of interferometric stability.

Window fouling is one of the most persistent problems in powder handling, fluidized-bed processing, fermentation, tobacco production and other processes involving dust, droplets or adhesive residues (Wrobel et al., 2020). As the deposit thickens, the centre-burst amplitude and integrated single-beam energy generally decrease together. A reduction of less than approximately 10%–15% relative to a clean-window reference may initially be managed by collecting a new background spectrum, provided that fringe modulation, spectral repeatability and prediction residuals remain within validated limits. A decline of approximately 15%–25% indicates that physical cleaning, such as pulsed air purging or mechanical wiping, should be initiated. When the interferogram amplitude or single-beam energy decreases by more than approximately 25%–30%, particularly when accompanied by a signal-to-noise loss greater than 30%–40%, quantitative output should be withheld until the optical path has been restored. These ranges are proposed as initial engineering criteria; the actual limits depend on detector dynamic range, source intensity, spectral resolution, optical geometry and the required analytical accuracy (Raza et al., 2026).

Mechanical vibration is especially important because conventional Fourier-transform instruments derive spectral information from precisely measured changes in optical path difference. Vibration from motors, conveyors, fans or fluidized-bed blowers can perturb moving-mirror velocity, alter the sampling position of the interferogram and introduce phase errors. Under clean conditions, rapid scanning, laser-referenced sampling and scan averaging may suppress part of this disturbance. Once fouling has weakened the interferogram, however, the same absolute vibration produces a larger relative error. The resulting spectrum may exhibit peak broadening, apparent wavenumber displacement or reduced repeatability. In practice, consecutive-scan variation should be monitored together with reference-laser stability and phase residuals. An increase of more than approximately 20%–30% in short-term spectral variance relative to the validated baseline may indicate inadequate vibration isolation or unstable optical-path sampling. Appropriate measures include corner-cube retroreflectors, vibration-tolerant interferometer geometries, rigid optical mounting, isolation pads, separation from rotating machinery and averaging of several rapid scans rather than a single long acquisition (Jin et al., 2025).

Background mismatch constitutes a second major source of systematic error. Absorbance is commonly calculated using a background spectrum collected before the sample measurement. If the window, source or atmospheric optical path changes after background acquisition, the background and sample no longer represent the same instrumental state. A contaminated sample spectrum divided by a clean background can produce broad pseudo-absorption, baseline oscillation or local negative absorbance. Recollecting the background may reduce these artefacts, but it is valid only when the remaining optical throughput is adequate. Repeatedly updating the background under severe fouling can conceal the visible baseline shift without recovering sensitivity to the sample. Background renewal should therefore be permitted only when the center-burst amplitude, single-beam energy and peak-to-noise ratio remain above predefined acceptance limits (Debus et al., 2019).

Temperature variation affects several components simultaneously. Changes in source temperature modify radiant output, detector temperature alters responsivity and dark signal, and thermal expansion may affect interferometer alignment and optical path. Temperature variation can also change the phase behaviour of the interferogram. For high-precision online applications, detector or optical-response drift of 1%–2% may already be comparable with the spectral change caused by a small variation in moisture or composition. Thermal stabilization, enclosure temperature control and temperature-dependent correction models should therefore be applied. Background and reference measurements should be collected after thermal equilibrium rather than immediately after instrument start-up (Kadam et al., 2010). If wavenumber position, phase and spectral intensity drift simultaneously with enclosure temperature, the disturbance should be attributed to thermal or interferometric instability rather than to the process material.

For open-path and mid-infrared systems, fluctuations in atmospheric water vapour and carbon dioxide may add narrow absorption structures that are unrelated to the sample. These bands are particularly problematic when the ambient optical path differs between the background and sample measurements. Dry-air or nitrogen purging, shorter exposed optical paths and masking of strongly affected regions can reduce this interference. Source ageing and detector drift must also be separated from window contamination. If single-beam energy decreases while the reference-laser signal, wavenumber repeatability and interferometer phase remain stable, source deterioration or window fouling is more likely. If energy loss occurs together with wavenumber displacement, phase instability and reduced scan repeatability, the fault is more likely associated with the interferometer, scanning mechanism or thermal environment (Schmitt, 2024).

The high-throughput advantage of Fourier-transform spectroscopy should not be interpreted as immunity to fouling. Greater initial optical throughput delays the point at which detector noise dominates, but it cannot reconstruct photons blocked or absorbed by a contaminated window. Similarly, increasing the number of co-added scans improves random noise approximately through averaging but cannot remove systematic background mismatch, phase distortion or wavelength-dependent attenuation. Excessive averaging may also reduce temporal resolution and conceal rapid process changes (Qin et al., 2025). The number of scans should therefore be selected by balancing signal-to-noise improvement against the dynamics of the production process.

A reliable online control strategy should operate at three diagnostic levels. The first level evaluates the interferogram through centre-burst amplitude, modulation depth, peak-to-root-mean-square-noise ratio and phase stability. The second evaluates the single-beam spectrum through integrated energy, background deviation, atmospheric bands and scan-to-scan repeatability. The third assesses the quantitative model through prediction bias, RMSEP and applicability-domain statistics. A reduction of approximately 10%–15% in interferogram amplitude can be used as an early warning for background renewal; a decline of 15%–25% should trigger cleaning; and a loss exceeding 25%–30%, a signal-to-noise retention below approximately 60%–70%, or a prediction bias greater than twice the validated RMSEP should result in rejection of the analytical output. Through this hierarchical approach, fouling-induced attenuation can be distinguished from true interferometer malfunction, and physical maintenance can be initiated before an apparently stable numerical prediction becomes analytically invalid (Wang et al., 2018). Table 6 is represent Main Online Interferences and Stabilization Measures for Fourier-Transform Spectrometers.

**TABLE 6:** Main online interferences and stabilization measures for fourier-transform spectrometers.

Interference source	Quantitative manifestation	Diagnostic indicator	Recommended measures	Engineering threshold
Window fouling	Interferogram peak amplitude and single-beam energy decrease 10%–25%; SNR decreases ∼20%	Interferogram amplitude, integrated spectral energy, peak-to-noise ratio	Background recollection, pulsed air purge, mechanical cleaning	Mild 10%–15%; Moderate 15%–25%; Severe >25–30%
Mechanical vibration	Increased relative error in weak signals; interferogram peak broadening	Moving mirror velocity, phase error, scan-to-scan variance	Vibration-tolerant interferometer, corner-cube retroreflector, mechanical isolation, rapid co-added scans	Peak variance increase 20%–30% above baseline
Background mismatch	Pseudo-absorption, baseline fluctuation, local negative absorbance	Comparison between sample and background spectra	Recollect background	Background mismatch due to background aging
Insufficient optical flux	SNR decreases even with high source throughput	Single-beam integrated energy, SNR	Suspend output, clean window	Peak drop >25–30% or SNR <60–70%
Interferometer malfunction	Deviations in wavenumber, phase, and repeatability simultaneously	Reference laser, interferogram phase, scan repeatability	Maintenance, scanning mechanism calibration	Multiple instrumental indicators exceed control limits
Model invalidation	Output appears stable, but interferogram abnormal	Prediction bias, RMSEP, applicability-domain statistics	Suspend prediction and inspect instrument	Bias > RMSEP warning; >2×RMSEP reject output

The values in the table are provided as engineering guidance and should be validated for each specific spectrometer, optical configuration, and industrial operating condition.

### 4.4 Multi-parameter coupled drift and compensation in online AOTF spectroscopy

AOTF spectrometers select wavelengths electronically by varying the radio frequency applied to an acousto-optic crystal. Their non-mechanical wavelength-selection mechanism provides rapid switching and relatively high resistance to mechanical vibration, making them suitable for high-speed online measurements. However, AOTF performance is jointly affected by the optical source, RF driver, acousto-optic crystal, detector and process-facing optical path. Unlike conventional filter-based instruments, in which disturbances are often manifested mainly as intensity drift, a single disturbance in an AOTF system may simultaneously alter wavelength position, diffraction efficiency, output intensity, spectral bandwidth and beam direction.

Window contamination is one of the primary sources of online measurement error. Dust, condensed moisture, oil films and adhesive residues reduce the optical throughput reaching the crystal and detector (Bogomolov et al., 2018). Because AOTF diffraction efficiency is wavelength dependent, the degree of fouling cannot be reliably inferred from the absolute intensity of a single channel. For example, a 15% decrease in one channel may arise from window contamination, source degradation, RF-power drift or a change in crystal diffraction efficiency. If attenuation differs among selected wavelengths, false variations in channel ratios may occur even when the actual process composition remains unchanged. As provisional engineering ranges, a reference-channel loss below 10% may be considered mild, 10%–20% may be treated as a warning condition, and a loss exceeding 20%–30% may indicate severe contamination. An independent reference detector and a fouling-monitoring wavelength should therefore be incorporated. A stable reference signal accompanied by a simultaneous decline in process-facing channels suggests window contamination, whereas proportional decreases in both reference and sample channels indicate source or RF-drive instability.

Crystal-temperature drift is a characteristic interference in AOTF systems. Temperature changes modify both the acoustic velocity and refractive index of the crystal, thereby altering the relationship between RF frequency and selected wavelength. Diffraction efficiency and spectral bandwidth may also vary simultaneously. For a system with a nominal resolution of 5 nm, a wavelength displacement equal to 10%–20% of the resolution corresponds to approximately 0.5–1.0 nm. Such a shift may already produce a significant quantitative error when the selected wavelength lies on the steep edge of an absorption band. Continuous crystal-temperature monitoring and a temperature-dependent RF–wavelength correction model are therefore required. The RF electronics should also be thermally controlled because, in high-precision applications, an RF-power or channel-intensity drift of approximately 1%–2% may be comparable to the response associated with a small change in moisture or concentration (Gonzaga and Pasquini, 2005).

RF-frequency instability and RF-power instability produce different effects. Frequency variation primarily causes wavelength displacement, whereas power variation primarily changes diffraction efficiency and output intensity (Chen et al., 2025). If intensity changes while the wavelength standard remains stable, RF-power or source drift is more likely. If wavelength shifts while intensity remains relatively stable, the problem is more likely related to frequency calibration. Simultaneous intensity and wavelength changes indicate coupled thermal instability of the crystal or RF driver. A critical-channel drift greater than approximately 1%–2% should therefore trigger intensity normalization or recalibration.

Sequential scanning is another major limitation of AOTF online measurements. Although wavelength switching is rapid, a complete spectrum is still assembled from wavelengths acquired at different times. When particles or fibers move rapidly through the optical footprint, different wavelengths may correspond to different particles or spatial locations, producing a temporally assembled pseudo-spectrum. Extending integration time to compensate for signal loss improves the signal-to-noise ratio but aggravates temporal averaging and inter-channel mismatch. As an engineering objective, the complete wavelength cycle should be shorter than 10% of the characteristic process-variation period. For example, if the material state changes substantially within 100 ms, the key wavelength sequence should ideally be completed within approximately 10 ms. If the coefficient of variation of repeated spectra or predictions increases by more than 20%–30% relative to the validated baseline, the acquisition cycle may be too slow (Schulte et al., 2026). Corrective measures include shortening integration time, reducing the number of selected wavelengths, interleaving key wavelengths and synchronizing acquisition with conveyor or particle-flow speed.

AOTF systems are also affected by beam-pointing and polarization changes. Temperature variation or optical misalignment can shift the diffracted beam at the detector or fiber interface. Even when wavelength selection remains accurate, reduced coupling efficiency may appear as intensity drift, particularly when a small detector aperture or fine-core fiber is used. This sensitivity can be reduced by enlarging the collection aperture, improving mechanical fixation, monitoring beam position and stabilizing polarization (Zhao et al., 2013).

Limited spectral redundancy creates an additional risk. Some AOTF instruments measure only a few characteristic wavelengths to maximize speed, reducing their ability to distinguish window contamination, source drift and genuine compositional variation. A more robust configuration should include analytical wavelengths, reference wavelengths, at least one fouling-monitoring wavelength and periodic wavelength-validation points. Model validity should also be assessed using reference-channel stability, wavelength residuals, short-term repeatability and applicability-domain statistics. A reference-channel decline greater than approximately 15%, a wavelength error reaching 10%–20% of nominal resolution or a prediction bias exceeding the validated RMSEP should trigger recalibration or inspection. A bias exceeding twice the RMSEP, or simultaneous failure of intensity, wavelength and model-validity checks, should result in suspension of the reported output.

A reliable AOTF compensation strategy should therefore operate at three levels. The first stabilizes the hardware through crystal-temperature control, RF-frequency and RF-power monitoring, source referencing and rigid optical alignment. The second corrects the signal using wavelength-resolved intensity normalization, temperature-dependent wavelength compensation and repeated reference channels within the scanning sequence. The third validates the result using prediction bias, short-term repeatability and model-applicability checks. Mild drift may be corrected when reference loss remains below 10%, wavelength error remains below 10% of nominal resolution and prediction bias remains within RMSEP. A reference loss of 10%–20% or a wavelength error approaching 10%–20% of the resolution should trigger recalibration and cleaning. Signal loss greater than 20%–30% or prediction bias above twice RMSEP should result in output rejection. Table 7 is represent main online interferences and compensation measures for AOTF spectrometers.

**TABLE 7:** Main online interferences and compensation measures for AOTF spectrometers.

Interference source	Quantitative manifestation	Diagnostic indicator	Recommended measures	Engineering criterion
Window fouling	Unequal attenuation among channels; reference-channel loss of 10%–30% or more	Reference detector, fouling-monitoring wavelength and channel consistency	Pulsed air purge, window cleaning and wavelength-resolved normalization	Mild <10%; warning 10%–20%; severe >20–30%
Crystal-temperature drift	Simultaneous wavelength shift and diffraction-efficiency change	Crystal temperature, wavelength standard and channel intensity	Active thermal control and temperature-dependent RF–wavelength correction	Wavelength error reaches 10%–20% of nominal resolution
RF-power drift	Intensity changes while wavelength remains nearly stable	Reference detector and RF-power monitor	Power normalization and RF-driver stabilization	Critical-channel drift >1–2%
RF-frequency drift	Selected wavelength deviates from its nominal value	Wavelength standard and RF-frequency monitor	Frequency recalibration and closed-loop stabilization	Wavelength residual exceeds the validated limit
Sequential-scanning mismatch	Different wavelengths correspond to different particles or spatial positions	Cycle-to-cycle variance and channel-ratio stability	Shorten scanning cycle, interleave wavelengths and synchronize with material motion	Full cycle preferably <10% of the process-variation period
Excessive integration time	Higher SNR but greater temporal averaging	SNR, response delay and prediction CV	Shorten integration time and average multiple rapid complete cycles	CV increases by 20%–30% above baseline
Beam-coupling drift	Intensity decreases while source and wavelength remain stable	Beam position and fiber-coupling signal	Increase collection aperture, stabilize optics and monitor beam position	Coupling loss exceeds the validated tolerance
Limited spectral redundancy	Difficulty distinguishing contamination from true compositional change	Consistency among analytical, reference and fouling channels	Add reference, fouling-monitoring and wavelength-validation channels	Diagnostic indicators become abnormal despite stable predictions

The numerical ranges of 10%–30%, 1%–2%, and a scanning cycle shorter than 10% of the process-variation period are provisional engineering criteria. They should be validated for the specific AOTF, spectrometer, optical configuration, process material and industrial operating conditions.

## 5 Technical issues and prospects of online infrared spectrometers

Currently, the four mainstream technical routes—filter-based, acousto-optic tunable filter (AOTF), grating dispersive, and Fourier transform infrared spectroscopy (FTIR)—inevitably encounter three core technical challenges in industrial environments: instrument stability, spectral data acquisition and processing, and hardware and software compatibility. As Process Analytical Technology advances, the paradigm has continuously transitioned from offline and at-line detection toward real-time and adaptive monitoring; however, innovations in online infrared spectroscopy have approached the physical limits of traditional optics. First, there exists an inverse relationship between instrument stability and measurement precision, where enhancing one often compromises the other. Second, discrepancies arise between results derived from discrete data gaps caused by incomplete acquisition and those obtained from comprehensive spectral information. Third, a misalignment persists between the physical limits of instrument hardware and the coordination of algorithmic models.

**FIGURE 13:**
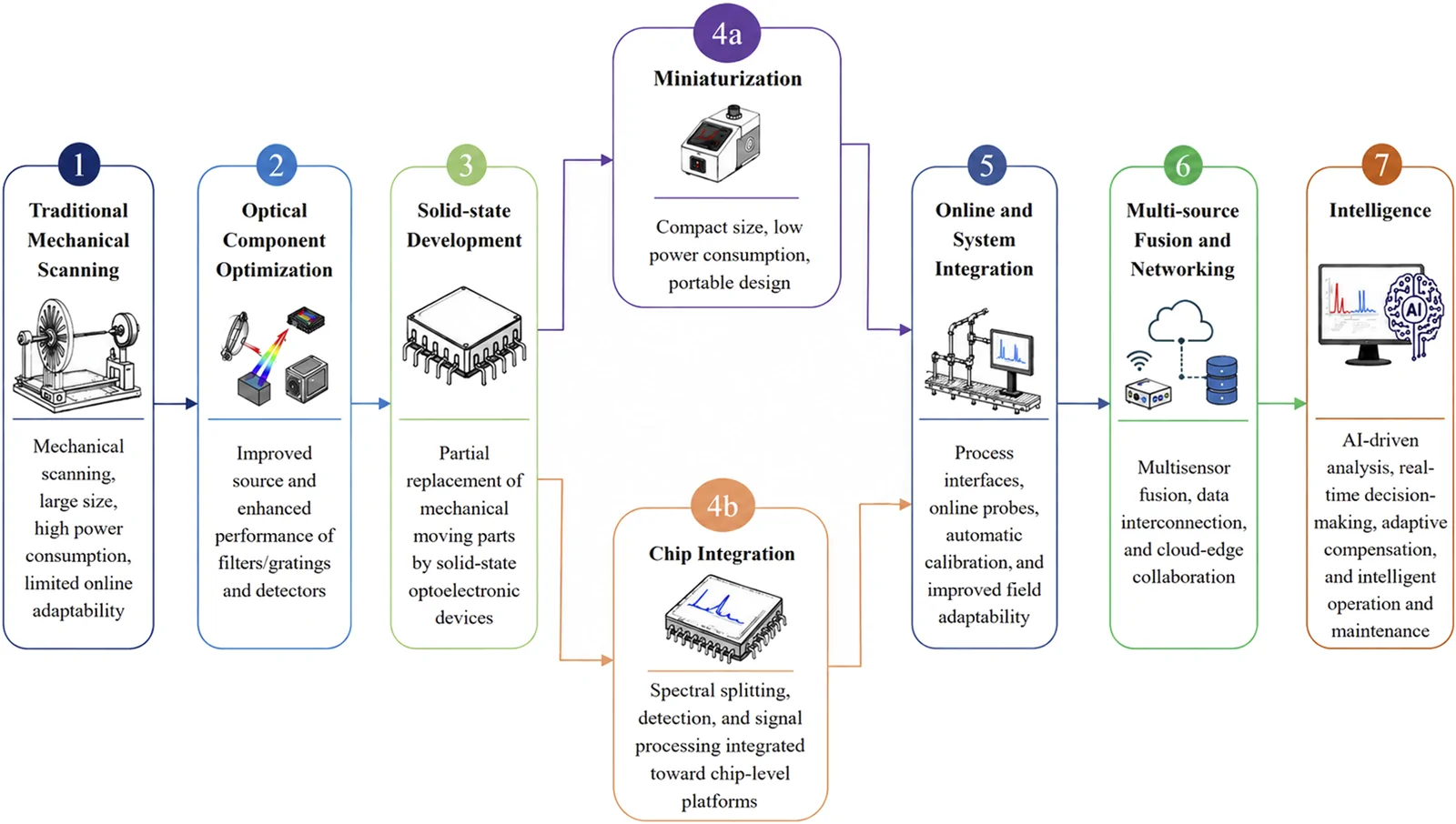
Evolution of online infrared spectroscopy technology.

Regarding technological evolution and future trends, given the inherent physical constraints of instrumentation and the low compatibility with complex industrial sites, the advancement of online infrared spectroscopic instruments can no longer be confined to the performance enhancement of single components. Instead, it necessitates a holistic optimization of technological architectures. A deep integration of multi-dimensional technologies—including MEMS, silicon photonic integration, high-dimensional spatiotemporal imaging, and computational spectroscopy—is essential. This convergence will drive the continuous renewal and expansion of online measurement techniques, spectroscopic methodologies, and application scenarios. Figure 13 illustrates the evolutionary direction of online infrared spectroscopy technology.

### 5.1 Discussion on the problems and bottlenecks of online spectroscopy technology

In terms of hardware, existing technological architectures fundamentally struggle to achieve a balance between high resolution and high stability, thereby exposing complex vulnerabilities during dynamic detection in industrial environments (Gałuszka et al., 2015; Wiesent et al., 2010). For grating scanning instruments and FTIR spectrometers, the mechanical moment of inertia inherent in stepper motors and precision moving mirrors imposes a ceiling on spectral acquisition rates while rendering systems highly sensitive to vibration. Consequently, during the monitoring of high-velocity fluids, the lag in mechanical scanning time induces spectral distortion, such that a single spectrum fails to represent the transient compositional content of the material under identical conditions. In the progression toward solid-state designs, while AOTF utilizes acousto-optic crystals to achieve fully solid-state tuning, the sensitivity of their birefringence to temperature gradients causes wavelength drift. Conversely, filter-based technology, although possessing superior stability, necessitates the sacrifice of spectral continuity.

Regarding data dimensionality, the ideal measurement condition for traditional infrared spectrometers relies on single-point hypothetical analysis, erroneously assuming that the spot coverage area represents the analytical result for the entire material volume. This assumption leads to significant deviations, particularly when characterizing highly heterogeneous materials such as ores and cut tobacco. Filter-based instruments acquire only a few specific discrete wavelengths, resulting in the loss of baseline drift information and complex frequency modulation data. Calibration is thus restricted to least squares or linear correction methods, which diminishes the model’s anti-interference capability. While grating array spectrometers can obtain complete and continuous spectra, the requisite InGaAs detectors are prohibitively expensive, necessitating a compromise between spectral resolution and spatial resolution (Lux et al., 2022).

At the algorithmic level, traditional chemometrics establishes models via linear regression; however, in the context of hardware miniaturization and non-standardized application scenarios, these models face risks of failure and inaccurate information resolution. Traditional Fourier transform algorithms are incapable of correcting or mitigating measurement deviations arising from hardware defects. This geometric increase in the difficulty of obtaining comprehensive and accurate data means that differences across technological routes, manufacturers, production batches, and production lines can cause deviations in high-precision model calibration, escalating maintenance and transfer costs. Although miniaturization, intelligence, and integration represent the evolutionary direction of online spectral analysis, the absence of standardized constraints regarding waveguide dispersion and manufacturing tolerances in the core technologies of miniaturized spectrometers exacerbates interferogram nonlinearity and degrades resolution, ultimately compromising the accuracy of material composition measurement (Allegrini and Olivieri, 2011; Pan et al., 2025).

### 5.2 Development trends and prospects of online spectroscopy technology

Driven by advancements in chip technology and artificial intelligence, the requisites for infrared spectrometers have transcended mere structural simplification and enhanced precision, evolving from precision optomechanical instruments into adaptive, intelligent sensing nodes within production lines. Future spectroscopic instrumentation is irrevocably transitioning from precision optics toward miniaturization, solid-state integration, and intelligence. The technical trajectory involves utilizing MEMS and metasurface technologies to integrate detectors onto silicon substrates, achieving arbitrary modulation of the light field through electrically controlled phase regulation. This approach not only resolves issues regarding the vibration resistance of spectroscopic instruments but also eliminates the physical aberrations and thermal drift responses associated with traditional instrumentation, thereby facilitating their integration into production lines for multi-angle, multi-line deployment and intelligent perception of material components.

As hardware performance enhancements drive physical properties to their ultimate limits, eventually reaching theoretical thresholds in sensitivity, response time, and measurement accuracy, algorithmic models and the elevation of sensing dimensions can be leveraged to analyze and regulate the precision of material information. Furthermore, cloud-based data can be utilized to implement self-adaptive regulation and calibration of instruments, thereby reducing maintenance costs and enhancing production line efficiency. Regarding the dimension of sensing, spectroscopic instruments must evolve from single-point measurement toward the fusion of hyperspectral imaging and ultrafast time-resolution technologies, forming a four-dimensional monitoring process. This capability allows for real-time feedback on the average content of material components while continuously generating thermal distribution maps of constituents, enabling the instrument to visualize chemical kinetic processes and monitor the stability of transient responses under extreme environments and processing conditions (Huck et al., 2021; Zavala-Ortiz et al., 2022).

Beyond hardware innovations, future industrial spectroscopy relies heavily on advanced algorithmic paradigms to ensure long-term model sustainability and trust. On the model level, integrating adaptive calibration and continual learning prevents performance decay during domain shifts, while explainable AI (XAI) and uncertainty quantification (UQ) provide transparent reasoning and predictive confidence bounds for automated control. Furthermore, hybrid mechanistic-data-driven modeling embeds physical laws to improve generalization beyond empirical training ranges. On the system level, coupling sensing nodes with digital twins and federated learning enables real-time virtual-physical synchronization and privacy-preserving knowledge aggregation across distributed manufacturing sites.

## 6 Summary

To transition process analytical spectroscopy from localized successes to universal industrial deployment, vague calls for “multi-faceted optimization” are insufficient. The research community must pivot towards establishing concrete, actionable frameworks. First, there is an urgent need for standardized physical testing protocols. Future regulatory guidelines (building upon frameworks such as ASTM or the recent ICH Q14) must define standardized experimental procedures to explicitly quantify the vibration sensitivity, thermal drift coefficients, and probe fouling tolerance of different optical architectures under controlled, simulated industrial stresses (Borman et al., 2025). This would allow practitioners to objectively compare the ruggedness of FTIR, AOTF, and grating instruments beyond ideal laboratory specifications.

Second, the chemometric and deep learning communities must embrace open science and public benchmark datasets. Currently, algorithmic innovations are validated on fragmented, proprietary datasets, making comparative performance claims unverifiable. The field requires publicly accessible “multi-stress” spectral datasets that capture controlled physical interferences (e.g., dynamic particle size changes and ambient temperature fluctuations). Finally, to curb the proliferation of poorly validated artificial intelligence models, journals and societies must establish minimum reporting requirements for deep learning in spectroscopy. Studies claiming superiority over traditional methods should be strictly required to report performance based on independent external batches (e.g., Leave-One-Batch-Out validation), transparently disclose network parameter counts, and provide mandatory baseline comparisons against thoroughly optimized linear models (e.g., PLS or EMSC-PLS) (Coca-Lopez et al., 2025; Heil et al., 2021). Only through such rigorous, standardized guidance can the practical impact of online spectroscopic monitoring be fully realized.
